# Bacterial chemotaxis in a microfluidic T-maze reveals strong phenotypic heterogeneity in chemotactic sensitivity

**DOI:** 10.1038/s41467-019-09521-2

**Published:** 2019-04-23

**Authors:** M. Mehdi Salek, Francesco Carrara, Vicente Fernandez, Jeffrey S. Guasto, Roman Stocker

**Affiliations:** 1 Ralph M. Parsons Laboratory, Department of Civil and Environmental Engineering, Massachusetts Institute of Technology, Cambridge, MA 02139 USA; 2 Institute for Environmental Engineering, Department of Civil, Environmental and Geomatic Engineering, ETH Zurich, 8093 Zurich, Switzerland; 3 Department of Mechanical Engineering, Tufts University, 200 College Avenue, Medford, MA 02155 USA

**Keywords:** Bacteria, Microbial ecology

## Abstract

Many microorganisms have evolved chemotactic strategies to exploit the microscale heterogeneity that frequently characterizes microbial habitats. Chemotaxis has been primarily studied as an average characteristic of a population, with little regard for variability among individuals. Here, we adopt a classic tool from animal ecology – the T-maze – and implement it at the microscale by using microfluidics to expose bacteria to a sequence of decisions, each consisting of migration up or down a chemical gradient. Single-cell observations of clonal *Escherichia coli* in the maze, coupled with a mathematical model, reveal that strong heterogeneity in the chemotactic sensitivity coefficient exists even within clonal populations of bacteria. A comparison of different potential sources of heterogeneity reveals that heterogeneity in the T-maze originates primarily from the chemotactic sensitivity coefficient, arising from a distribution of pathway gains. This heterogeneity may have a functional role, for example in the context of migratory bet-hedging strategies.

## Abstract (web summary)

Chemotaxis is usually considered as a trait of a species or population. Here, the authors use a microfluidics device to reveal that clonal *E*. *coli* show individual variation in a key aspect of chemotactic behavior.

## Introduction

The habitats of microorganisms are often rich in chemical gradients, whether in oceans^1^, soil^2^, or the human body^3^. Swimming cells have evolved advanced sensing strategies to exploit chemical gradients, and the resulting process—chemotaxis—allows them to move towards favorable conditions or away from harmful ones. The life of a motile microorganism is thus often a sequence of gradient-navigation events, each representing a chemotactic decision of how rapidly the cell moves up or down the gradient.

Chemotaxis is a fundamental behavioral strategy in a range of unicellular biological systems, including cancer cells during metastasis^4^, spermatozoa migrating toward an egg^5^, and neutrophils moving toward inflammation or infection sites^6^. Among bacteria, examples of chemotaxis are many and diverse, ranging from *Escherichia coli* homing in on multiple chemoattractants^7^, to *Helicobacter pylori* migrating toward the mucus lining of the stomach^8^, to *Vibrio cholerae* swimming toward the intestinal mucosa^9^, to *Vibrio coralliilyticus* chemotaxing toward the mucus of its coral host^10^, to multiple species of marine bacteria swimming toward dissolved organic matter^11,12^. The chemotactic performance of a population also differs greatly among species. Marine bacteria, for example, typically exhibit higher chemotactic velocity and tighter chemotactic accumulation than enteric bacteria^13^.

Chemotaxis has largely been regarded as an average characteristic of a population. However, it has now become clear for many biological functions that—even in the absence of genetic variation or environmental cues—intracellular biochemical noise, arising from stochastic gene expression and partitioning of proteins and mRNA at cell division, can induce the differential expression of proteins and functional molecules among cells^14–18^. Such purely phenotypic heterogeneity, or nongenetic diversity^19^, has been demonstrated in a number of microbial systems, for processes ranging from growth^20^ to attachment^21^. Among these, one important example of cell-to-cell variation in the distribution of functional parameters is heterogeneity in the amount of proteins involved in chemotaxis^22,23^, which could lead to a nonuniform response to chemoattractants of cells within a population. Having a distribution of phenotypes can be beneficial for bacterial populations, for example, in bet-hedging and division-of-labor strategies, or community self-regulation^24,25^.

Within the chemotaxis pathway of *E*. *coli*, one of the most extensively characterized chemosensory systems, there are several potential sources of nongenetic diversity. These include the number of transmembrane chemoreceptors that bind ligands and repress the autophosphorylation of the protein CheA; the messenger proteins CheY and CheZ, which regulate the flagellar switching rate from counter-clockwise to clockwise by phosphorylation and dephosphorylation; and the proteins CheR and CheB, which regulate the adaptation time by altering the sensitivity of the receptors via methylation and demethylation, respectively^7^. Variability in the expression of proteins within the chemotaxis pathway is expected to generate a distribution of chemotactic performances within a population by affecting multiple traits that contribute to chemotactic performance^19^. Three fundamental phenotypic traits are the tumble bias, which measures the probability of a cell tumbling, the pathway gain, which determines how strongly a cell perceives and amplifies a given gradient, and the adaptation time, which measures the timescale needed for the chemotactic machinery to rescale the value of the gradient over the background concentration^19,26^. The tumble bias, together with the swimming speed, characterizes the random walk in the absence of a chemical gradient, and determines the diffusivity of the bacteria. All these traits contribute to the chemotactic velocity, which is the average drift of the cells in the direction of a chemical gradient. The chemotactic sensitivity, which is the coefficient of proportionality between the chemotactic velocity and the gradient^27^, is a combined measure of the chemotactic performance. Recent experiments^28^ demonstrated that heterogeneity in the tumble bias induces differential migration of clonal *E*. *coli* cells along a gradient. However, how sources of heterogeneity in multiple other phenotypic traits within a population affect the distribution of the chemotactic sensitivity coefficient among cells is not well understood. Here, we address this gap and show how nongenetic diversity in the chemotactic sensitivity coefficient affects the chemotactic migration of cells in different sensing regimes, namely the linear-sensing regime, when cells respond to the absolute value of the gradient, and the logarithmic-sensing regime, when cells respond to the gradient rescaled by the absolute concentration^29,30^.

We quantify the degree of heterogeneity in the chemotactic sensitivity coefficient within a clonal bacterial population by using a new microfluidic device. The device has a branching maze geometry that allows the spatial sorting of the better chemotaxers from within a population while simultaneously assessing their chemotaxis properties. Branching maze geometries have been widely used in ecology, including to test chemical preferences in birds^31,32^, maze navigation, decision-making, and learning in nematodes^33^, collective behavior in microbes^34^, chemotaxis to organic chemicals in slime molds^35^, and the routing of plant roots in response to volatile chemicals^36^. In many cases, the fundamental geometrical elements of a maze are Y- or T-junctions, which demands a binary choice from individual organisms, and the distribution of choices within a population can then be assessed by counting the organisms within each arm of the junction.

Inspired by the classic T-maze often used in ecological studies, we designed an iterative microfluidic T-maze to study the chemotactic decision-making of bacteria and specifically to quantify the variability in the chemotactic sensitivity coefficient within a population. In the maze, bacteria are faced with a series of consecutive encounters with a gradient, implemented as a series of four consecutive T-junctions. Each T-junction necessitates a decision by the bacteria, consisting of the migration up or down the gradient of chemoattractant at the junction. The likelihood that an individual bacterium will successfully navigate the gradient and make the correct decision depends on the sensitivity of its chemotaxis pathway. Using single-cell video microscopy and image analysis, we quantified the behavior of hundreds of cells at each junction by measuring the relative number of cells in the up or downgradient portion of the junction. A mathematical model accounting for phenotypic heterogeneity captures the fundamental sorting mechanism and provides a quantitative characterization of heterogeneity in the chemotactic sensitivity coefficient among individual cells. Comparison of the distribution of cells among consecutive junctions reveals that the chemotactic abilities of cells are heterogeneous even within a clonal population, and that our T-maze is able to sort the highly chemotactic cells from within a heterogeneous population.

## Results

### Microfluidic T-maze assay

**Fig. 1:**
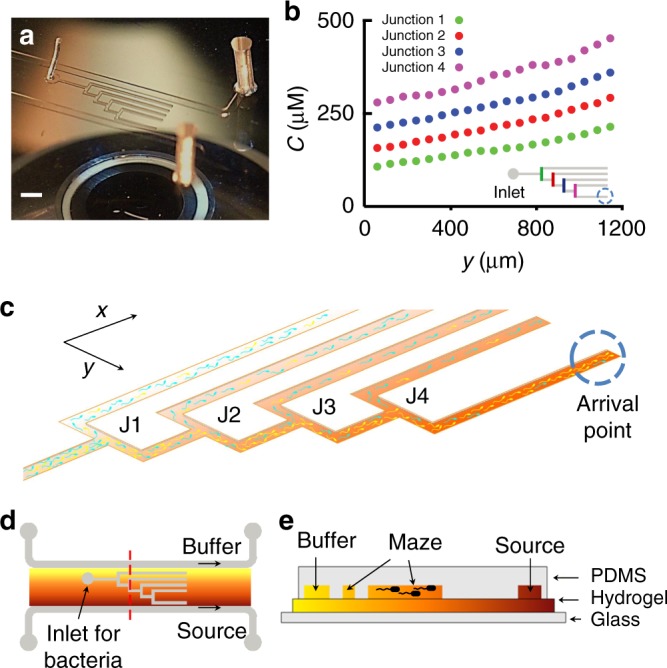
The microfluidic T-maze. **a** A microfluidic T-maze device. The scale bar shown in the figure is 2 mm. **b** By using source and buffer channels running parallel to the maze to generate a steady chemoattractant gradient within the hydrogel base, the device creates concentration gradients that have the same magnitude through consecutive junctions (here shown for log-sensing). **c** Schematic of a mixed population of cells swimming through the maze. Better chemotaxers (yellow) become increasingly concentrated at sequential junctions. **d** Schematic of the device and **e** cross-section (corresponding to the red dashed line in (**d**)). Flow of the chemoattractant through the source channel generates a gradient in the hydrogel, and hence also along each T-junction section (vertically oriented channel segments in (**d**)) of the maze channel. A droplet containing cells is placed at the inlet shown in (**d**), and cells swim from left to right through the maze, making multiple chemotactic decisions through the consecutive junctions

The microfluidic T-maze consists of a sequence of T-junctions overlaid on a background gradient of a chemoattractant (Fig. 1; Methods). Each T-junction comprises a microchannel segment oriented perpendicular to the chemoattractant gradient that branches into two microchannel segments parallel to the chemoattractant gradient, where cells face the decision of swimming up or down the gradient (Fig. 1). The operational principle of the maze is that, at a T-junction, chemotactic bacteria statistically bias their swimming preferentially in the direction of the gradient, so that a fraction of the population greater than half will move into the branch of the T-junction that harbors a higher concentration of chemoattractant. However, since the chemotaxis process has a stochastic component, a fraction of cells will still migrate into the other branch. The proportion of cells entering the branch of the T-junction with higher chemoattractant concentration represents a measure of the strength of the population’s chemotactic sensitivity coefficient.

To sort chemotactic cells in the face of this inherent stochasticity, the microfluidic maze—unlike traditional T-mazes^31,32^ —comprises four T-junctions arranged in series. At each subsequent T-junction, the absolute concentration of the chemoattractant is higher, but the concentration gradient is the same for all T-junctions (Fig. 1b). Cells reaching the branch with the highest chemoattractant concentration after the fourth T-junction will have made the correct decision four times. The distribution of cells at each junction is used to quantify the chemotactic sensitivity coefficient of that subpopulation of cells and then compared to other junctions.

### Chemotactic cell sorting of a mixed population

**Fig. 2:**
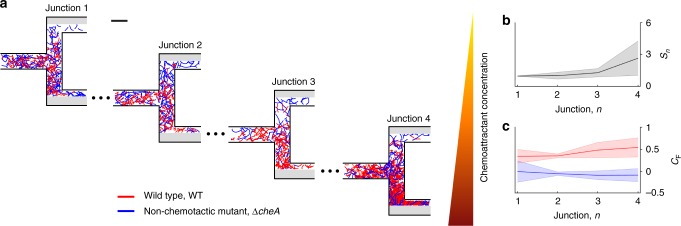
Demonstration of cell sorting in the microfluidic T-maze using marine bacteria. **a** The microfluidic T-maze sorts *Marinobacter adhaerens* wild type (red) from a nonchemotactic mutant (Δ*cheA*; blue) in an initial 1:1 mixture as cells migrate through the device. Chemoattractant concentration (liquid 2216 medium) increases from top to bottom in the figure (yellow–red color scale). Diagrams of the junctions show measured cell trajectories. The scale bar shown in the figure is 200 μm. **b** The sorting index, *S*_*n*_, the ratio of WT to mutant cells found at each junction. **c** The choice factor, *C*_F_, measuring the tendency of cells to swim up the gradient at each junction (red and blue for wild type and mutant, respectively). Curves in (**b**) and (**c**) show mean ± s.d. over three replicates

To demonstrate the principle and efficacy of the microfluidic T-maze in sorting cells by chemotactic sensitivity coefficient, we first performed experiments with a mixture of two strains of *Marinobacter adhaerens*, a motile marine bacterium that uses chemotaxis to home in on phytoplankton exudates^37^. A wild-type strain (WT) and a mutant strain deficient in chemotaxis (Δ*cheA-*cfp) were injected into the maze at a 1:1 concentration ratio. At each junction, WT cells demonstrated a clear preference for the chemoattractant (liquid 2216 medium; Methods), consistently migrating more strongly into the branch of the T-junction containing higher chemoattractant concentration (Fig. 2a). In contrast, Δ*cheA-*cfp cells divided in equal proportions between those migrating up and down the gradient, resulting in sequential dilution by a factor of two at each subsequent junction (Fig. 2a).

The difference in the chemotactic behavior of the two strains was quantified by computing a choice factor, *C*_F_ = (*N*_UP_ − *N*_DOWN_)/(*N*_UP_ + *N*_DOWN_), where *N*_UP_ and *N*_DOWN_ are the numbers of cells observed in the branch of the T-junction having the higher and the lower chemoattractant concentration, respectively. The choice factor ranges from −1 to +1 and is proportional to the tendency of cells to swim up the gradient, with 0 indicating no preference. We found that *C*_F_ was positive (0.43 ± 0.16) for WT cells and approximately zero (−0.05 ± 0.14) for Δ*cheA-*cfp cells (mean ± s.d. over three experimental replicates; Fig. 2c). The behavioral difference between the two strains led to sorting of the WT strain, whose concentration increased with distance into the positive branch of the maze relative to that of the nonchemotactic mutant, as a result of the stronger dilution of the latter at each junction (Fig. 2). This sorting is captured by a second metric, the sorting index *S*_*n*_, defined as the ratio of the number of WT cells to the number of Δ*cheA-*cfp cells at a given junction, *n*. The sorting index was normalized to 1 in junction 1 (to account for any small differences in the initial cell concentration of the two strains) and was found to progressively increase to 2.6 ± 1.5 in junction 4 (Fig. 2b), denoting an increasing ratio of WT to mutant cells in subsequent junctions.

### Chemotactic cell sorting within a clonal population

Having demonstrated that the microfluidic T-maze sorts chemotactic cells in a mixed population, we then used it to investigate and quantify the heterogeneity in the chemotactic sensitivity coefficient of cells belonging to a clonal population. For these experiments we used *E. coli* (HCB 33), because of the extensive characterization of chemotaxis in this species at the population level since the seminal work of Berg^38^ and of the availability of established mathematical models of chemotaxis^39^. Chemotactic sorting experiments were performed using a gradient of methylaspartate (MeAsp), a nonmetabolizable analog of aspartate and a known attractant for *E*. *coli*. Two gradients were tested, one ranging between 0 and 5 µM MeAsp and one between 0 and 500 µM MeAsp (the numbers indicate the level of the chemoattractant for the buffer and the source), to capture two different chemotactic regimes. In the lower-MeAsp regime (0–5 µM), cells are in the linear-sensing regime^29,30^ and respond to the absolute value of the chemical concentration gradient, $\nabla$*C* (which is constant throughout our maze). In contrast, in the higher-MeAsp regime (0–500 µM), cells are in the logarithmic-sensing regime^29,30^, and respond to the relative magnitude of the chemical gradient, $\nabla$*C*/*C*, i.e., to the MeAsp gradient rescaled by the MeAsp concentration, *C* ($\nabla$*C*/*C* decreases in successive junctions of the maze).

**Fig. 3:**
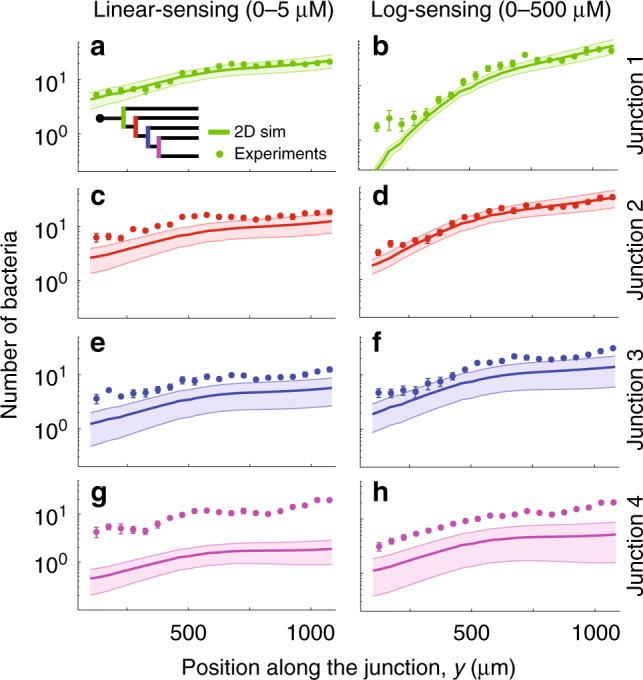
Accumulation profiles of bacteria at the four maze junctions for a monoclonal *E*. *coli* population responding to a methylaspartate gradient. Panels show the absolute number of bacteria observed at points along each T-junction (circles, mean ± s.d. from three replicates) in the linear-sensing (**a**, **c**, **e**, **g**) and log-sensing (**b**, **d**, **f**, **h**) regimes. Lines show the expected number of bacteria from the simulations. The shaded confidence interval shows the variation in the simulation solution over the time interval of the experiments (40–160 min). Bacteria are introduced at the inlet in the maze at *t* = 0 min (see Fig. 1) and disperse over time into the maze (see Supplementary Fig. 2 for the accumulation profiles at two different time points). The chemotactic sensitivity in the simulations was fitted to best match the experimental profiles at junction 1. Higher numbers of bacteria are found at later junctions in the maze compared to the simulations performed with a chemotactically homogeneous population of cells, as a consequence of the sorting mechanism

We quantified the profile of cell concentration, *B*(*y*) (with *y* being the direction of the gradient), along each junction of the maze for both the linear-sensing and the log-sensing regimes (Fig. 3). Cell concentration profiles are skewed in the direction of the gradient for both regimes, denoting positive chemotaxis, yet more so in the log-sensing regime because of the 100-fold higher levels of chemoattractant. Furthermore, in the log-sensing regime (but not in the linear-sensing regime) the cell distribution skewed less consistently from junction 1 to 4 (Supplementary Fig. 1). This decrease is in agreement with theoretical expectations, owing to the decrease in $\nabla$*C*/*C* in subsequent junctions (Methods).

To determine the presence and magnitude of heterogeneity in the chemotaxis of individual cells within the population, we compared the experimentally determined bacterial concentration profiles in the four junctions with the results from two-dimensional numerical simulations of the bacterial transport equation (performed in COMSOL Multiphysics; Methods), in the same geometry as in the experiments. In the simulations, the diffusion component models the random motility of bacteria (with diffusion coefficient^40^ *D* = 330 μm^2^ s^−1^), while the advection component models the chemotactic bias of cells toward the chemoattractant. The advection term is implemented through a chemotactic velocity, *v*_c_, with *v*_c,G_ = *χ*_G_$\nabla$*C* in the linear-sensing regime and *v*_c,L_ = *χ*_L_$\nabla$*C*/*C* in the log-sensing regime (Methods). Here, *χ*_G_ and *χ*_L_ are the chemotactic sensitivity coefficient for the linear-sensing and log-sensing regimes, respectively (not to be confused with the receptor sensitivity^41^, Methods). The average chemotactic sensitivity coefficients of the entire population were determined by fitting the bacterial concentration profile predicted from the numerical simulations to the experimentally observed distribution at junction 1 (Supplementary Fig. 1a, b), separately for the linear-sensing and the log-sensing regimes (Methods). The fit was accurate for both regimes (Fig. 3a, b and Supplementary Fig. 1a, b), indicating that the correct functional form of the cell distribution profiles is predicted by the simulations at junction 1, and yielding *χ*_G_ = 6.4 × 10^2^ μm^2^ s^−1^μM^−1^ and *χ*_L_ = 1.4 × 10^3^ μm^2^ s^−1^ (note that we here call both *χ*_G_ and *χ*_L_ chemotactic sensitivity coefficients despite them having different units).

Using these values of the full-population-averaged chemotactic sensitivity coefficient to predict the cell distribution in junctions 2–4 with the model, we found a consistent deviation between the predicted and the observed cell distribution profiles. In particular, a progressively greater number of bacteria in later junctions accumulated up the gradient in the experiments compared to the simulations (Fig. 3), for both the linear-sensing and the log-sensing regimes (Supplementary Fig. 1) and across multiple time points (Supplementary Fig. 2). This discrepancy points to heterogeneity in the chemotactic sensitivity coefficient of the population, whereby cells reaching subsequent junctions do not represent a uniform subsample of the full population at junction 1, but a subpopulation with a higher chemotactic sensitivity coefficient than the average.

To quantify the strength of the chemotactic response at each junction, we estimated a metric proportional to the chemotactic velocity, *v*_c_, which measures the speed at which cells move up a gradient (Supplementary Fig. 1). These estimates were obtained by using the relation between *v*_c_ and the slope of the bacterial concentration profile, *B*(*y*). For a one-dimensional gradient, the steady-state solution of the bacterial transport equation (Methods) yields *B*(*y*) = *B*_0_ exp[(*v*_c_/*D*)*y*]. We thus compared the slope of log [*B*(*y*)/*B*_0_] vs. *y*, which at steady-state would be equal to *v*_c_/*D*, between experiments and simulations. Since the experiments did not reach steady state, at each T-junction we estimated the slope of the distribution only in the portion of the channel between 750 and 1100 μm (Supplementary Fig. 1, Supplementary Table 1), which corresponds to the section of the channel running up the gradient. This choice was made to obtain an estimate of the slope from the exponential scaling of the concentration profile, away from the middle of the junction where the diffusive flux of bacteria over the *x*-direction creates an accumulation in the bacterial profiles. This accumulation tends to dissipate over time (Supplementary Fig. 2) as the flux dissipates (at steady state the equilibrium solution converges to the exponential profile). For the linear-sensing regime, the slope of log [*B*(*y*)/*B*_0_] (calculated over the branch leading up the gradient, see Methods for details) in the simulations decreased more than 20-fold from junction 1 (8.8 ± 0.4 × 10^−4^ μm^−1^) to junction 4 (0.35 ± 0.07 × 10^−4^ μm^−1^), whereas in the experiments the opposite trend was found, with the slope increasing more than 5-fold from junction 1 (4.5 ± 1.5 × 10^−4^ μm^−1^) to junction 4 (25 ± 3 × 10^−4^ μm^−1^).

The increase in the slope of the bacterial concentration profile (i.e., the higher value of *v*_c_/*D*) is caused by cells with a higher chemotactic sensitivity coefficient—and not by cells with a lower diffusivity—reaching subsequent junctions. Given that the diffusivity *D* is proportional to *v*^2^*τ*_0_, where *v* is the swimming velocity and *τ*_0_ is the typical reorientation time^42^, a cell can modulate its diffusivity by changing its velocity or its reorientation time. Tracking cells swimming in the T-maze revealed variation in the swimming velocity of the seeding population at the inlet (Supplementary Fig. 3), with faster cells reaching later junctions at earlier times. Yet, after an initial transient, the mean swimming speeds for the cells within the four junctions were observed to converge to the same value (Supplementary Fig. 3), whereas the spatially skewed distributions of cells caused by chemotaxis were preserved over time along the junctions (Supplementary Fig. 2). The increase in the swimming speed observed at later junctions (Supplementary Fig. 3) could be induced by an increase in the reorientation time *τ*_0_ or by an increase in the run speed *v*_r_ (see Supplementary Discussion). The early wave of cells that show a higher velocity could in general be a product of either a higher run speed or a lower tumble bias (the latter would be in agreement with previous results^28^).

Our detection of a positive change in the bacterial concentration profile and hence in the chemotactic sensitivity coefficient must be a product of an increase in the pathway gain (Supplementary Fig. 1). We note that the chemotactic sensitivity coefficient still depends on the swimming speed, on the adaptation time, and on the tumble bias, but we do not expect these dependences to affect our conclusions. First, changes in swimming speed would not affect the slope of the bacterial concentration profile, because the chemotactic sensitivity coefficient and the diffusivity have the same quadratic dependence on the swimming speed, and the slope is determined by their ratio (Methods, Eqs. (3)–(7)). Physiological variation in the swimming speed generates a distribution of diffusivities. Numerical simulations accounting for this distribution of diffusivities yielded a minor decrease in the slope of the bacterial concentration profile in subsequent junctions, compared to a population with a single, average diffusion coefficient (Supplementary Fig. 4, Supplementary Table 2). Second, in our model we assumed that cells have a constant adaptation time that is smaller than the residence time of cells in the junction (~150 s) and greater than the run time (which is in the order of 1 s). This assumption is supported by typical values of the adaptation time^43,44^ ranging from 1 to 30 s. Under such conditions, variations in the adaptation time are rather inconsequential for the chemotactic velocity (see Supplementary Discussion). Third, an increase in the run time (i.e., a decrease in the tumble bias) would also not change the slope of the bacterial concentration profile, as revealed by Eq. (7) (Methods). These considerations strongly indicate that the bacteria that reached higher-order junctions in our experiments were more chemotactic—as a result of increasing the pathway gain—compared to the average of the entire population at junction 1. The T-maze platform thus revealed heterogeneity in the distribution of the pathway gain among a bacterial population, a result that is complementary to recent work reporting heterogeneity in the distribution of the run time or the tumble bias^28^. Through our two-dimensional branching design we could quantify the slope of the bacterial concentration profile at each junction (something that is not possible to obtain with previously used one-dimensional experimental assays^28^), and thus determine the relative change in the phenotypic traits of cells reaching each junction by disentangling their relative contributions to the slope. Together, the combination of recent results and ours show that multiple sources contribute to phenotypic heterogeneity in chemotactic sensitivities of individual cells within a population of bacteria.

In contrast to the increase in the slope of log [*B*(*y*)/*B*_0_] in the linear-sensing regime, in the log-sensing regime, the slope decreased almost 30-fold in the simulations (22 ± 1 × 10^−4^ μm^−1^ in junction 1 to 0.8 ± 0.4 × 10^−4^ μm^−1^ in junction 4), but remained approximately constant in the experiments (17 ± 4 × 10^−4^ μm^−1^ in junction 1 to 18 ± 3 × 10^−4^ μm^−1^ in junction 4) (Supplementary Fig. 1, Supplementary Table 1). An analysis of covariance of the slopes among junctions confirmed that there was no difference among the slopes for the log-sensing regime (ANOVA, *F*_3,20_ = 0.18; *P* = 0.91), whereas there was a difference among the slopes for the linear-sensing regime (*F*_3,20_ = 18.4; *P* < 10^−4^). The difference in the trends between the two sensing regimes derives from the differences between the chemotactic velocity in these regimes, which for any given cell is predicted to remain constant across subsequent junctions in the linear-sensing regime as a result of the constant gradient (*v*_c,G_ = *χ*_G_$\nabla$*C*), but to decrease in the log-sensing regime (*v*_c,L_ = *χ*_L_$\nabla$*C*/*C*), in view of the increase in *C* (Fig. 1b). The near-constancy across consecutive junctions of the slope of log [*B*(*y*)/*B*_0_] in the log-sensing regime thus arises from the compensatory effect of the decrease in $\nabla$*C*/*C* and the increase expected from the selection of more strongly chemotactic cells in subsequent junctions, whereas the selection of these cells is apparent on the background of the constant gradient in the linear-sensing regime.

**Fig. 4:**
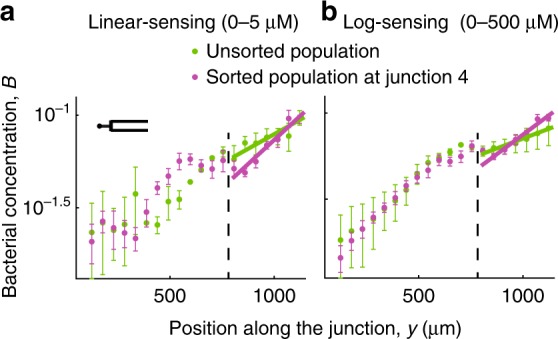
Accumulation profiles of *E*. *coli* from an unsorted and a sorted population. The accumulation profile of *E*. *coli* from the unsorted population (green) in the single-junction device is compared to the accumulation profile of the sorted population (purple) at the last junction (junction 4) in the T-maze for the linear-sensing (**a**) and log-sensing (**b**) regime. The gradient generated over the single-junction device corresponds to that in the last junction of the T-maze. The fitted slope of the bacterial accumulation profile in the portion of the junction up the gradient (to the right of the dashed line) is shown for each case. Figures show mean ± s.d. over two (unsorted) or three (sorted) replicates

To provide direct evidence for population sorting, we also compared the behavior of an unsorted population in a gradient corresponding to that in the last junction (junction 4) of the T-maze with the behavior of sorted bacteria in junction 4. In this single-junction experiment (Supplementary Fig. 5), the cells were exposed from the onset to a level and gradient of chemoattractant equal to that occurring in junction 4 of the full T-maze experiment. The results show a higher slope in the bacterial profile for the sorted population (i.e., the full T-maze experiment) compared to the unsorted population (i.e., the single-junction experiment) (Fig. 4), for both the log-sensing regime (18 ± 3 × 10^−4^ μm^−1^ vs. 9 ± 0.8 × 10^−4^ μm^−1^) and the linear-sensing regime (2.5 ± 0.3 × 10^−3^ μm^−^^1^ vs. 1.4 ± 0.1 × 10^−3^ μm^−1^). An analysis of covariance of the slopes between sorted and unsorted populations confirmed that there was a statistically significant difference between the slopes for the two populations for both log-sensing (*F*_1,10_ = 8.29; *P* = 0.02) and linear-sensing regimes (*F*_1,10_ = 8.35; *P* = 0.02). This confirms that the cells that reached junction 4 in the T-maze experiments had indeed been sorted, i.e., they had a chemotactic performance above the average of the unsorted (i.e., initial) population.

Taken together, these results indicate the existence of phenotypic heterogeneity in chemotaxis in *E*. *coli*, with a distribution of chemotactic sensitivity coefficients among individual cells of the population. Thus, while the effective chemotactic sensitivity coefficient averaged over the full population in junction 1 was *χ*_G_ and *χ*_L_ for the linear-sensing and log-sensing regimes, respectively, the sorting of the better chemotaxers along the maze resulted in an increased effective chemotactic sensitivity coefficient of the fraction of the population arriving at subsequent junctions.

### Quantification of chemotactic heterogeneity

**Fig. 5:**
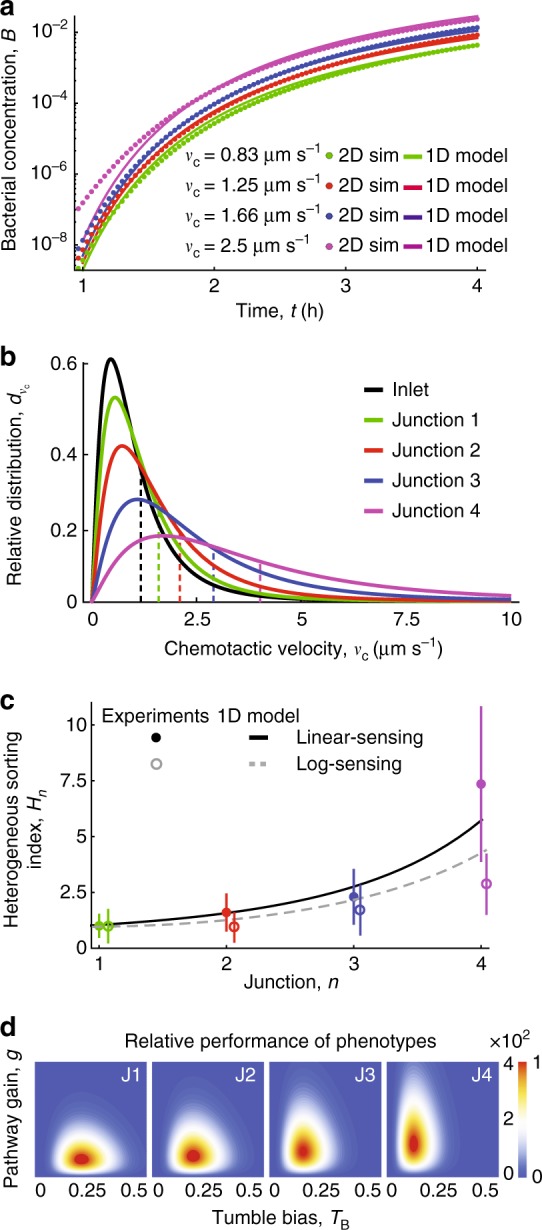
Cell sorting of a chemotactically heterogeneous population of bacterial cells. **a** Predictions from a 1-D advection-diffusion model at junction four (lines) match the results from 2-D simulations based on the actual maze geometry (circles), for four values of chemotactic velocity within the expected range for *E*. *coli* (0.8–2.5 µm s^−1^). **b** Based on the analytical model of chemotactic heterogeneity, the relative distribution of chemotactic velocities in a heterogeneous population of cells shifts to higher values in consecutive junctions of the maze. Dashed vertical lines show the mean of each distribution. **c** The heterogeneous sorting index is the ratio of the expected number of cells at each junction from a heterogeneous population compared to those from a homogeneous population with the same initial mean chemotactic velocity. Filled circles show the mean *H*_*n*_ over the three experimental replicates for the linear-sensing regime (0–5 μM) and open circles for the log-sensing regime (0–500 μM). Error bars are ± s.d. over the three experimental replicates. The solid black and dashed gray lines are the predictions from the 1-D advection-diffusion model for the linear-sensing and log-sensing regimes, respectively. **d** Relative performance of phenotypes (from lower performance in blue to high performance in red) as a function of pathway gain and tumble bias for a monoclonal population of cells reaching each junction in the T-maze (junctions 1–4 from left to right)

To quantify the magnitude of the variability in the chemotactic sensitivity coefficient within the population, we developed an analytical model of maze navigation that accounts for phenotypic heterogeneity by varying both tumble bias and pathway gain. The model allowed us to study how these two phenotypic traits, which affect the diffusivity and chemotactic sensitivity coefficient, contribute to the experimentally observed cell concentration profiles and chemotactic performances in the maze junctions (Methods, Supplementary Fig. 6). By working in coordinates directed along the individual branching channels, the model collapses the two-dimensional geometry of the maze to a one-dimensional (1-D) formulation (Methods; Supplementary Fig. 7) that is amenable to an explicit analytical prediction of the number of bacteria present at each junction over time, as a function of the distribution of chemotactic sensitivity coefficients in the population. In this model, the spreading of the population through the maze consists of a diffusion solution along the maze segments perpendicular to the chemoattractant gradient and an advection-diffusion solution along the segments parallel to the gradient. The 1-D model was successfully validated by comparison with numerical simulations in the 2-D branching geometry (Fig. 5a, Supplementary Figs. 6a, 8) for a chemotactically homogeneous population (in which each cell had the same swimming speed and chemotactic sensitivity coefficient).

In the absence of chemotaxis, when only the random motility of bacteria (with diffusivity *D*) is considered, the expected number of bacteria after junction *n* is computed as the solution to a diffusion equation (Methods), yielding *B*_*n*_(*t*) = $\frac{{B_0}}{{2^n}}\frac{{{\mathrm{e}}^{ - d_n^2/(4Dt)}}}{{\sqrt {4{\mathrm{\pi }}Dt} }}$, where *B*_0_ is the initial concentration of bacteria and *d*_*n*_ is the one-dimensional distance from the inlet to that junction (Supplementary Fig. 7). Chemotaxis can be incorporated into this solution by multiplying *B*_*n*_ by a sorting index,

1 $$S_n = {\mathrm{e}}^{ - \mathop {\sum }\nolimits_{i = 1}^n \frac{ iy_vv_{c_i}}{{4D}}}{,}$$

which represents the ratio of the advection-diffusion solution and the diffusion solution (see Methods for derivation), calculated at the end of junction *n*, where *y*_*v*_ is the length of the semibranch of each junction in the *y*-direction. Note that the predicted exponential increase of the sorting index *S*_*n*_ with the junction number *n* (Eq. (1)) is in qualitative agreement with the increase of the sorting index observed in the experiments with *M*. *adhaerens* (compare Fig. 2b with Supplementary Fig. 6b). The exponential dependence of the ratio of the two populations on the junction number in Eq. (1) indicates that sorting could be further improved by increasing the number of junctions in the device.

We used the model to predict how chemotactic heterogeneity affects the spatial distribution of cells and the navigation performance in the maze (Eq. (9)), compared to a chemotactically homogeneous population. We considered a population having a distribution $f_{g,T_{\mathrm{B}}}$ of pathway gains, *g* (Eq. (10), and tumble bias, *T*_B_ (Eq. (11), Supplementary Fig. 9). This translates into a distribution of chemotactic velocities with mean $\bar v_{{\mathrm{c}}_0}$ (the number in subscript indicates the junction considered, with 0 being the inlet) (Eq. (12), Fig. 5b, Methods). For the homogeneous population, the chemotactic velocity was taken to be equal to ${\bar{v}}_{{\mathrm{c}}_0}$. Note that in the linear-sensing regime, working in terms of chemotactic velocity is equivalent to working in terms of chemotactic sensitivity coefficient, because there is a linear relationship between the two, i.e., *v*_c,G_ = *χ*_G_$\nabla$*C* (the gradient is a constant). We then used the model to compare the number of cells reaching the end of the maze with the highest chemoattractant concentration for the two populations. To compute the navigation performance throughout the maze—and thus understand how the maze sorts cells with different chemotactic sensitivity coefficient—we multiply the diffusion solution *B*_*n*_ by the sorting index (Eq. (9), Methods). For the linear-sensing and log-sensing regimes, this comparison yielded 7.4-fold and 2.9-fold more cells of the chemotactically heterogeneous population in junction 4 compared to the homogeneous population (Fig. 5c, Methods), showing that the shift in the distribution of chemotactic velocities towards higher values deeper into the maze (Fig. 5b) produces a greater accumulation of cells in the highest-concentration regions. In the linear-sensing regime, cells reaching the end of the maze (junction 4) had considerably higher chemotactic velocities than the average of the entire population at the inlet. Cells arriving at consecutive junctions in the maze are predicted to have substantially higher mean values of the chemotactic velocity (${\bar{v}}_{{\mathrm{c}}_{1},{\mathrm{G}}}$ = 1.1 µm s^−^^1^, ${\bar{v}}_{{\mathrm{c}}_{2},{\mathrm{G}}}$ = 1.4 µm s^−1^, ${\bar{v}}_{{\mathrm{c}}_{3},{\mathrm{G}}}$ = 2.1 µm s^−1^, ${\bar{v}}_{{\mathrm{c}}_{4},{\mathrm{G}}}$ = 3.3 µm s^−1^; Fig. 5b).

The model allowed us to reconstruct the distribution of phenotypes as a function of pathway gain and tumble bias for cells reaching each junction in the T-maze (Fig. 5d) and to estimate the chemotactic heterogeneity in the pathway gain of the population for the two different regimes. To this end, we fitted the ratio between the number of cells reaching the end of each junction for the chemotactically heterogeneous population from the experiments and the chemotactically homogeneous population from the simulations (Fig. 3) to the prediction of the analytical model. In this fitting, the only free parameter was the width of the distribution of the pathway gains for the full population. By combining experimental data with the model, we find that variation in both tumble bias and pathway gain affect the navigation performance in the T-maze (Methods). Cells are selected for both lower tumble bias and higher pathway gain at later junctions in the T-maze. However, cells with low tumble bias are predominantly selected at earlier time points, whereas the selection of cells with high pathway gain is stable over time (Supplementary Fig. 10). This selection is reflected in a shift towards higher values of the distribution of chemotactic velocity. In addition, we find that both tumble bias and pathway gain contribute to the increase in the sorting index, but that the pathway gain is the main contributor to the increased slope observed at later junctions in our experiments (Supplementary Discussion). In summary, we conclude that the chemotactic sensitivity coefficient, arising from the pathway gain, is the predominant contributor to the heterogeneity in chemotactic performance that we observed in the T-maze.

## Discussion

Heterogeneity in the biological functions of bacteria may considerably impact their response to environmental stimuli as well as the collective performance of a population. Our results show that a wide spectrum of chemotactic sensitivity coefficients are maintained within a clonal population of *E*. *coli*, suggesting considerable levels of intrinsic noise in the expression of the surface receptors or proteins that are part of the chemotaxis pathway^7^, in line with recent theoretical findings^19^.

Intrapopulation heterogeneity in the climbing of chemical gradients might be beneficial for a clonal population of bacteria. Given the fact that microbial motility is a balance between exploring (diffusion) and exploiting (chemotaxis) of chemical landscapes, heterogeneity in chemotaxis implies that a population will have a spectrum of functionalities ranging from explorers to exploiters. This diversity will increase the chances of the population responding optimally to a given chemoattractant gradient. Better chemotaxers will be the first responders to ephemeral hotspots of nutrients, such as those occurring in the gut microenvironment for *E*. *coli*^45^ or in seawater for marine bacteria^1^. Less strongly chemotactic cells will remain more evenly distributed in space, potentially decreasing the impact of catastrophic events such as localized chemical insults or predation. More broadly, these results suggest that bet-hedging strategies—where a fraction of the population performs less well in a specific task but is adapted to alternative environmental conditions—could be important in chemotaxis^46^, as it is already known to be important in quorum sensing and biofilm formation^47^, the evolution of virulence in pathogens^25^ and antibiotic persistence^48^.

Our observations of heterogeneity in the chemotactic sensitivity coefficient, arising from a distribution of pathway gains, combined with the recently reported heterogeneity in the tumble bias^28^, demonstrates that multiple elements of chemotactic behavior in *E*. *coli* are subject to nongenetic heterogeneity. The two sources of heterogeneity—in tumble bias^28^ and pathway gain (this work)—pertain to different biological functions and have specific ecological relevance, as also predicted theoretically^19^: the tumble bias is most relevant in the response of cells to unsteady sources, whereas the pathway gain is most relevant in accumulation around sources in steady gradients. In this work, we have focused on the heterogeneity in pathway gain, isolating it from the effects of motility through the use of sorting junctions: while fast swimmers with low-tumble bias will navigate the maze more quickly, their sorting still occurs on the basis of their enhanced chemotactic sensitivity coefficient, rather than simply their speed.

Methodologically, the microfluidic T-maze device has potential applications in chemotactic cell sorting for a broad range of environmental, medical, and industrial applications. For example, in spermatozoa, chemotactic ability and swimming speed are correlated with fertility^49^, making methods to sort cells based on these functions desirable compared to labor-intensive conventional microscopy methods. In this respect, microfluidics provides a powerful alternative, in particular the range of microfluidic chemotaxis assays that in recent years have enabled the generation of steady chemical gradients by eliminating flow through the use of hydrogels^50^, as used here. Existing one-dimensional microenvironments for bacteria^28^ and spermatozoa^51^ segregate cells according to their motility characteristics (e.g., tumble bias^28^ and swimming speed^51^). The T-maze, in contrast, provides a noninvasive approach for purifying a population, because nonchemotactic cells are progressively diluted out, junction after junction. This mechanism results in the sorting and physical separation of the stronger chemotaxers along the maze, so that the accumulation of cells in the last branch of the maze provides sorting on the basis of both motility and chemotactic sensitivity coefficient.

Through microscale approaches such as the T-maze, our ability to detect and quantify heterogeneity among single cells within microbial populations will continue to be enhanced in traits from growth^20^ to antibiotic resistance^48^ to navigation^28^. This understanding of intrapopulation variability in biological functions that to date have been investigated primarily at the population scale will be an important element in advancing our ability to mitigate the harm and harness the potential of diversity within the microbial world.

## Methods

### Bacterial cultures

For the *E*. *coli* used in this work, a clonal population was cultured from a frozen stock of *E*. *coli* HCB 33 (also known as RP437) by first inoculating a culture plate. A single colony was picked from the plate to inoculate 2 ml of tryptone broth (TB) medium for overnight culture at 30 °C on an orbital shaker (300 rpm). This culture solution was resuspended and diluted (1/100) in TB and incubated for ~4 h to reach midexponential phase (OD_600_ = 0.4). The cells were then washed twice by centrifuging at 2300*g* for 5 min and resuspended in motility medium (10 mM potassium phosphate, 0.1 mM EDTA, 1 μM methionine, 10 mM lactic acid, pH 7)^52^. Finally, the cells were kept at 4 °C for 15 min to stop growth before experiments.

Two *M. adhaerens* strains^37^, a WT strain labeled with yellow fluorescent protein and a chemotaxis-deficient mutant that constitutively expressed cyan fluorescent protein (Δ*cheA-*cfp), were also used. Each strain was inoculated onto 2216 agar plates from frozen stocks and incubated overnight at 30 °C. One colony was then picked for each strain and inoculated into liquid 2216 medium (5 ml) containing ampicillin (50 μg/ml) and incubated overnight at 30 °C on an orbital shaker (300 rpm). Prior to experiments, cells were checked under a microscope to confirm fluorescence. Cells were washed twice in filtered autoclaved seawater by centrifuging at 5000*g* for 1 min, then resuspended in 1 ml filtered autoclaved seawater. The growth of each strain was determined via spectrophotometry (OD_600_ = 0.7) and, from this, cells from the two strains were diluted in filtered autoclaved seawater and combined at approximately equal proportions before they were introduced into the maze.

### Microfabrication of the microfluidic T-maze

The microfluidic T-maze device was fabricated using soft lithography^53^. A mold for the maze geometry and the auxiliary source and buffer channels (Fig. 1) was fabricated with SU8 on a silicon wafer. Microfluidic channels were then created by casting polydimethylsiloxane (PDMS; Sylgard 184 Silicone Elastomer Kit, Dow Corning, Midland, MI) onto the mold. The cured PDMS was then removed and punched at the maze entrance to provide access for tubing.

To complete the device construction, the PDMS channel structure was placed onto a hydrogel (agarose) slab without bonding, so that each microchannel consisted of three PDMS walls (top and sides) and one agarose wall (base) (Fig. 1). The hydrogel layer was prepared from a 3% (wt/vol) solution of agarose (SeaKem^®^LE Agarose, Lonza) in buffer medium (consisting of motility medium for *E*. *coli* and filtered autoclaved seawater for *M*. *adhaerens*). This solution was heated in a microwave oven, then injected between two glass slides separated by a 1 mm silicone gasket at the edges, and allowed to cool. The PDMS slab was placed onto the agarose slab and held in place during the experiment by applying gentle pressure using clamps. Flow was driven through the source and buffer channels using a syringe pump in withdrawal mode to create negative pressure and thus avoid delamination.

### Experimental setup and imaging

The microfluidic T-maze consists of three parallel sections, with two parallel channels for the chemoattractant source (MeAsp for *E*. *coli* and 2216 medium for *M*. *adhaerens*) and buffer (consisting of motility medium for *E*. *coli* and filtered autoclaved seawater for *M*. *adhaerens*) and the maze test section located between them (Fig. 1). The three channels were separated from each other by thin layers of PDMS (≤300 μm). The chemoattractant diffuses through the underlying hydrogel from the source to the buffer, which creates a steady concentration field in the hydrogel (linearly decaying between source and buffer) and consequently in the test section.

In preparation for an experiment, the PDMS channel was placed in a vacuum chamber (−0.6 bar) for 6 min and the PDMS-on-hydrogel device was then filled with buffer medium (motility medium for *E*. *coli* and filtered autoclaved seawater for *M*. *adhaerens*). The chemoattractant gradient was allowed to develop through the device over 2.5–3 h in the absence of bacteria to allow time for diffusion from the source to the buffer channel. This time was sufficient to allow the concentration field to become established in the device, as verified experimentally by conducting tests using fluorescein (Supplementary Fig. 11). Each chemotaxis experiment began by placing a droplet of the cell suspension at the inlet and then inoculating the inlet port by using a thin syringe needle. Cells were then observed as they migrated through the maze. Each set of experiments was replicated three times. Experiments were conducted at room temperature.

Bacteria were imaged at the mid-plane of the device using phase contrast microscopy (10× objective; Andor Zyla camera with 6.5 µm/pixel; numerical aperture = 0.30; with additional magnification 1.5×) at 25 frames/s. At regular intervals, each junction in the maze was imaged and cells were tracked using image analysis routines based on intensity thresholding in custom, automated routines in MATLAB (The MathWorks). From the tracks, bacterial concentration profiles were computed at each junction.

### Numerical simulations

The behavior of *E*. *coli* in the T-maze was modeled with an advection-diffusion model, using the exact experimental geometry, with reflecting boundary conditions at the T-maze walls. The model reads

2 $$\frac{{\partial B}}{{\partial t}} = D{\nabla} ^2B - \nabla \cdot ({\boldsymbol{{v}}}_{\mathrm{c}}B){,}$$

where the first term on the right-hand side accounts for the diffusion part, and the second term corresponds to the chemotactic drift. The chemotactic velocity *v*_c_ is a function of the chemical concentration *C* and of its gradient $\nabla$*C*. The chemical concentration is a linear function of the *y*-direction, stationary over time, so that $\nabla$*C* is a constant. *B*(*x*, *y*, *t*) is the bacterial concentration in the maze over time.

By employing parabolic scaling techniques, a recent analysis showed that the chemotactic drift in a (shallow) linear gradient^54^ (but see refs. ^26,55^ for a similar derivation in exponential gradients), such as the one employed in our experiments, can be expressed as a function of the ligand concentration and gradient *K*(*C*, $\nabla$*C*) and the chemotactic sensitivity coefficient *χ*_0_

3 $$v_{\mathrm{c}} = \chi _0K(C){\mathrm{ }} = \chi _0(K_{\mathrm{A}}-K_{\mathrm{I}})\nabla C[(K_{\mathrm{A}} + C){\mathrm{ }}(K_{\mathrm{I}} + C)]^{ - 1}{,}$$

with (see Supplementary Discussion)

4 $$\chi _0 = {\mathrm{1/2}}v^2\left( {1-a_0} \right)g\tau _0 = {\mathrm{1/2}}v^2\left( {1-a_0} \right)\tau _{\mathrm{t}}g/T_{\mathrm{B}}{,}$$

where *v* is the swimming speed (more precisely, the run speed), *v*_c_ is the chemotactic velocity, *C* is the chemoattractant concentration (methylaspartate), *K*_I_ = 18 μM and *K*_A_ = 2.9 mM are the dissociation constants for inactive and active Tar receptors, *a*_0_ denotes the steady-state kinase activity, and *τ*_0_ = *τ*_t_/*T*_B_ is the average run time, where *T*_B_ is the tumble bias and *τ*_t_ is the tumbling time. The pathway gain is *g* = *NH*, where *H* is the motor amplification coefficient, and *N* is the cooperativity, reflecting the number of receptors per cluster. The velocity of a swimming cell can be expressed as

5 $$v = v_{\mathrm{r}}\left( {1 - T_{\mathrm{B}}} \right){,}$$

where *v*_r_ is the velocity during a run. In deriving Eq. (5) we are assuming that, because cells swim at low Reynolds numbers, the time required to accelerate from 0 to *v*_r_ after a tumble is negligible compared to *τ*_t_^56^. For a cell swimming in a run and tumble pattern, the effective diffusivity is

6 $$D = v^2\tau _0/[3\;(1-\left\langle {\cos \theta } \right\rangle )] = v^2\tau _{\mathrm{t}}/[3T_{\mathrm{B}}(1-\left\langle {\cos \theta } \right\rangle )]{,}$$

where the mean of the cosine of the reorientation angle between two successive runs is $\langle{\cos \theta }\rangle$ ~ 1*/*3 (ref. ^42^). In the following, we assume no variation in the run speed *v*_r_, and the tumble time *τ*_t_, taken to be equal to 0.2 s (ref. ^55^), and that all the variation in the diffusivity is due to changes in the tumble bias *T*_B_.

Importantly, for MeAsp concentrations such that *K*_I_ ≪ *C* ≪ *K*_A_, the equation for *v*_c_ reduces to *v*_c_ ≈ (*χ*_0_/*K*_A_)(*K*_A_ − *K*_I_)$\nabla$*C*/*C*. Therefore, in this regime the chemotactic velocity can be written as *v*_c,L_ = *χ*_L_$\nabla$*C*/*C*, which corresponds to the Keller–Segel formulation^57^ and exhibits the log-sensing property^30^ (the subscript L denotes the log-sensing regime). In this regime, the magnitude of the chemotactic response depends on (i) the strength of the gradient, $\nabla$*C* = Δ*C*/*W*, where Δ*C* is the difference between the concentration in the source and in the sink (the latter is often 0, since buffer is run in the sink) and *W* is the distance between the source and the sink, and (ii) the concentration of MeAsp in the T-maze channels, *C*(*x*, *y*). Conversely, for MeAsp concentrations such that *C* ≪ *K*_I_, both terms (*K*_I_ + *C*)^−1^ and (*K*_A_ + *C*)^−^^1^ are essentially independent of the concentration *C*, and the chemotactic velocity can be written as *v*_c,G_ = *χ*_G_$\nabla$*C*, which corresponds to linear-sensing (the subscript G denotes the linear-sensing regime). Note that the ranges 0–500 and 0–5 μM for the log-sensing and linear-sensing regimes express the ranges in terms of the values of the MeAsp concentration within the sink (here, buffer in both cases) and the source channels. The minimum and maximum levels of MeAsp experienced by the cells in the test channel under each regime are *C*_Lmin_ = 47 μM, *C*_Lmax_ = 469 μM, *C*_Gmin_ = 0.47 μM, and *C*_Gmax_ = 4.69 μM (Fig. 1b). The cells in the inlet channel experience an average concentration of MeAsp of *C*_L,0_ = 140 μM and *C*_G,0_ = 1.4 μM, for the log-sensing and linear-sensing regimes, respectively. The two chemotactic sensitivity coefficients *χ*_L_ and *χ*_G_ were fitted to match the bacterial concentration profile in the first junction for the two different regimes. The diffusion coefficient of *E*. *coli* cells^40^ was taken to be *D* = 330 μm^2^ s^−^^1^. We solved the two-dimensional partial differential equations (Eq. 2) with COMSOL Multiphysics (Burlington, MA), in both the log-sensing (0–500 μM) and the linear-sensing regimes (0–5 μM).

### Fitting the experimental profiles

In order to compare the bacterial concentration profiles between the experiments and the numerical simulations, we used the exact experimental geometry of the T-maze, with reflecting boundary conditions at the T-maze walls. For the initial conditions, a step function was used with bacterial concentration equal to 1 inside the inlet circle and 0 everywhere, which simulates the injection of a droplet of cell suspension at the inlet. We fitted the slopes of the logarithm of *B*(*x*, *y*, *t*) for the two regimes. This slope is related to the chemotactic velocity of the bacteria, *v*_c_, and their diffusion coefficient, *D*. Within any T-junction the solution of the advection-diffusion model (Eq. (2)) at steady-state is *B*(*y*) = *B*_0_ exp[(*v*_c_/*D*) *y*]. The slope of the logarithm of *B*(*y*)/*B*_0_ is therefore *v*_c_/*D*.

The chemotactic accumulation length *L*_c_ = *D/v*_c_, which is the inverse of the slope, reads (see Supplementary Equation (6) for the derivation in the Supplementary Discussion)

7 $${L_{\mathrm{c}} = 2{\mathrm{/}}3\left( {1-\left\langle {\cos \;\theta } \right\rangle } \right)^{ - 1}/\left\{ {\left( {K_{\mathrm{A}}-K_{\mathrm{I}}} \right)\nabla C\left( x \right)\left[ {\left( {K_{\mathrm{A}} + C\left( x \right)} \right)\left( {K_{\mathrm{I}} + C\left( x \right)} \right)} \right]^{ - 1}g\left( {1-a_0} \right)} \right\}{.}}$$

The experimental profiles were captured for each junction over time, for three replicate experiments, and their averages are shown in Fig. 3 and Supplementary Fig. 1.

### The sorting index

The diffusion solution for the bacterial concentration along the branches of the T-maze (Eq. (2)) was approximated by a linear superposition of 1-D diffusion solutions, *B*_*n*_ = $\frac{{B_0}}{{2^n}}\,\frac{{{\mathrm{e}}^{ - d_{\mathrm{n}}^2/(4Dt)}}}{{\sqrt {4{\mathrm{\pi }}Dt} }}$, where *B*_0_ is the initial concentration of bacteria, *n* is the junction number, and *d* is the distance calculated along the path to junction *n*, where the inlet is *d*_0_ = 0 (Supplementary Fig. 7). The term 2^*n*^ in the denominator accounts for the even split of the bacterial concentration at the *n*th junction. In this analytical approximation, the solution in each branch is independent of that in the other branches; in other words, in the limiting case of branches of infinite length in the *x*-direction one can neglect the effects of the reflecting boundaries at the end of the T-maze. The advection-diffusion solution along the branches of the maze was calculated at the end of each T-junction by considering the diffusion solution multiplied by a sorting index, *S*_*n*_ = exp[$\mathop {\sum }\nolimits_{i = 1}^n iy_{\mathrm{v}}v_{{\mathrm{c}}_i}$/(4*D*)], where $v_{c_{i}}$ is the chemotactic velocity along the *y*-direction parallel to the chemical gradient at junction *i*, *y*_v_ is the length of the semibranch of each junction in the *y*-direction. The sorting index represents the ratio of the advection-diffusion solution and the diffusion solution, calculated at the end of the *n*th T-junction of length *y*_v_.

This quantity, calculated at time *t*_n_^*^= $\mathop {\sum }\nolimits_{i = 1}^n iy_{\mathrm{v}}/v_{{\mathrm{c}}_i}$, is

8 $$S_n = \frac{{{\mathrm{e}}^{ - (ny_{\mathrm{v}} - v_{\mathrm{c}}t_n^ \ast )^2/(4Dt_n^ \ast )}}}{{\sqrt {4{\mathrm{\pi }}Dt_n^ \ast } }} \left( {\frac{{{\mathrm{e}}^{ - (ny_{\mathrm{v}})^2/(4Dt_n^ \ast )}}}{{\sqrt {4{\mathrm{\pi }}Dt_n^ \ast } }}} \right)^{ - 1}_{|t_n^ \ast = \mathop {\sum }\limits_{i = 1}^n iy_{\mathrm{v}}/v_{{\mathrm{c}}_i}} = {\mathrm{e}}^{ -\mathop {\sum }\nolimits_{i = 1}^n \frac{{ iy_{\mathrm{v}}v_{{\mathrm{c}}_i}}}{{4D}}}{.}$$

### Navigation performance in the T-maze

In the T-maze, the navigation performance, *P*_*n*_, up to junction *n* of a cell, characterized by diffusivity, *D*, and chemotactic velocity, *v*_c_, is

9 $$P_n = B_0\left( {4{\mathrm{\pi }}Dt} \right)^{ - 1/2}{\mathrm{e}}^{ - \frac{{d_n^2}}{{4Dt}}}S_n = B_0\left( {4{\mathrm{\pi }}Dt} \right)^{ - 1/2}{\mathrm{e}}^{ - \frac{{d_n^2}}{{4Dt}}}{\mathrm{e}}^{ -\mathop {\sum }\nolimits_{i = 1}^n \frac{{ iy_{\mathrm{v}}v_{{\mathrm{c}}_i}}}{{4D}}}{,}$$

where the first term is the solution of the diffusion equation in the T-maze and *S*_*n*_ is the sorting index up to the junction n. We note that *D* and *v*_c_ are functions of the two variables, tumble bias, *T*_B_, and pathway gain, *g* (see Eqs. (3) and (6)). In order to calculate the performance *P* of a cell based on its phenotype characterized by the variables *g*, *T*_B_, we need to know the joint probability density function $f_{g,T_{\mathrm{B}}}$ of the phenotypes for a monoclonal population of *E*. *coli*. If we assume that the two variables *g*, *T*_B_ are independent, we can decompose the function $f_{g,T_{\mathrm{B}}}$ into the independent distributions of the two random variables $h_g,b_{T_{\mathrm{B}}}$.

The ranges of variation of the two variables are *T*_B_ $\in$ [0, 1] and for *g* = *NH*, *N*, *H* $\in$ ${\Bbb R}^ +$. The distribution $b_{T_{\mathrm{B}}}$ can be modeled as a beta distribution ${\cal{B}}$(*T*_B_;*α*,*β*), where the two parameters *α* = 5 and *β* = 17, which determine the shape of the tumble bias distribution, are fitted from data available in the literature^28^. In order to derive the distribution of the pathway gains *h*_*g*_ we need to compound the sources of variation within *N* and *H*, since *g* = *NH*. We do this below.

Regarding the cooperativity, *N*, which reflects the receptor clusters’ size^58^, we inferred the distribution of the cooperativity by analyzing fluorescence resonance energy transfer experiments at the single-cell level in *E*. *coli*. These experiments revealed strong variability in the composition of receptors at the single-cell level in a monoclonal population due to nongenetic variability in the gene expression of chemotaxis molecules. The ratio of the two most abundant chemoreceptors, Tar/Tsr, was found to be an increasing function of the nutrient concentration^58,59^. Given the high number of receptors per cell^60^ (>10,000), based on the central limit theorem we assume that all the clusters have the same cooperativity number *N* and the same Tar/Tsr ratio. Within each cluster, we are interested in estimating the cooperativity of the Tar receptors, because the Tsr receptors are unlike to be active since the binding constants for the Tsr receptor to methylaspartate are considerably higher (~100 mM) than the chemoattractant values adopted in our experiments (*C* < 0.5 mM). We fitted the random variable Tar/Tsr ratio, *r* = *N/*(*N*_tot_ − *N*), with a Gamma distribution *G*(*r*;γ,δ) with parameters *γ* = 2.2*; δ* = 0.7 (data from Fig. 4 in ref. ^59^), where *N*_tot_ = *N* *+* *N*_Tsr_ is the total size of each cluster. We make the assumption that the distribution of the cluster size *N*_tot_ is a Poisson distribution $\wp$(*N*_tot_;*λ*) with mean *λ* = 9. The variable of interest is the number of Tar receptors per cluster, *N* = *rN*_tot_/(*r* *+* 1), which is itself a random variable by virtue of being a function of random variables.

Regarding the motor gain, *H*, recent experiments determined the motor gain at the single-cell resolution^61^ and found *H* = 20, in contrast to previous studies that estimated *H* = 10 (ref.^62^). Since the motor gain in each cell can vary depending on the number of FliM molecules, a component of the switch complex at the base of the flagellar motor, we model the motor gain *H* as a random variable with a Gaussian distribution ${\cal{F}}$(*r*;*μ*,*σ*) with mean *μ* *=* 15 and standard deviation *σ*, a free parameter of the model.

By compounding all the sources of variation in the pathway by the transform rule for random variables^63^, the distribution of the pathway gains *h*_*g*_ can be expressed as

10 $$h_g = \mathop {\sum }\limits_{k = 1}^\infty \mathop {\sum }\limits_{N = 1}^k \begin{array}{*{20}{c}} {\frac{{\lambda ^k{\mathrm{e}}^{ - \lambda }}}{{k!}}{\mathrm{e}}^{ \frac{-N}{{\delta \left( {k - N} \right)}}}\left( {\frac{N}{{k - N}}} \right)^{\gamma - 1}\frac{{\delta ^{ - \gamma }k}}{{\left( {N - k} \right)^2}}\frac{{{\mathrm{e}}^{\frac{{ - \left( {\frac{g}{N} - \mu } \right)^2}}{{2\sigma ^2}}}}}{{N\sqrt {2{\mathrm{\pi }}\sigma ^2} }}} \\ {} \end{array}{.}$$

The joint probability distribution $f_{g,T_{\mathrm{B}}}$ for a cell expressing the phenotype *g* and *T*_B_ reads (Figure S9)

11 $$\begin{array}{*{20}{l}} {f_{g,T_{\mathrm{B}}}} \hfill & = \hfill & {h_g{\cal{B}}(T_{\mathrm{B}};\alpha ,\beta )} \hfill \\ {} \hfill & = \hfill & {\mathop {\sum}\limits_{k = 1}^\infty {\mathop {\sum}\limits_{N = 1}^k {\frac{{\lambda ^k{\mathrm{e}}^{ - \lambda }}}{{k!}}{\mathrm{e}}^{ \frac{-N}{{\delta (k - N)}}}} } } \hfill \\ {} \hfill & {} \hfill & { \times \left( {\frac{N}{{k - N}}} \right)^{\gamma - 1}\frac{{\delta ^{ - \gamma }k}}{{(N - k)^2}}\frac{{{\mathrm{e}}^{\frac{{ - \left( {\frac{g}{N} - \mu } \right)^2}}{{2\sigma ^2}}}}}{{N\sqrt {2{\mathrm{\pi }}\sigma ^2} }}} \hfill \\ {} \hfill & {} \hfill & { \times \frac{{{\mathrm{\Gamma }}(\alpha + \beta )}}{{{\mathrm{\Gamma }}(\alpha ){\kern 1pt} {\mathrm{\Gamma }}(\beta )}}(1 - T_{\mathrm{B}})^{\beta - 1}T_{\mathrm{B}}^{\alpha - 1}} \hfill \end{array}{,}$$

with *α* = 5, *β* = 17, *γ* = 2.2, *δ* = 0.7, *λ* = 9, *μ* = 15, and *σ* a free parameter of the model.

The distribution of the chemotactic sensitivity coefficient, and thus the chemotactic velocity at the inlet $d_{v_{\mathrm{c}}}$, can be calculated as the ratio *g/T*_B_ of the two random variables *g*, *T*_B_

12 $$\begin{array}{*{20}{l}} {d_{v_{\mathrm{c}}}} \hfill & = \hfill & {\frac{{{\mathrm{\Gamma }}(\alpha + \beta )}}{{{\mathrm{\Gamma }}(\alpha ){\kern 1pt} {\mathrm{\Gamma }}(\beta )}}\mathop {\int}\limits_0^\infty {\mathop {\sum}\limits_{k = 1}^\infty {\mathop {\sum}\limits_{N = 1}^k {\frac{{\lambda ^k{\mathrm{e}}^{ - \lambda }}}{{k!}}{\mathrm{e}}^{ \frac{-N}{{\delta (k - N)}}}\left( {\frac{N}{{k - N}}} \right)^{\gamma - 1}} } } } \hfill \\ {} \hfill & {} \hfill & { \times \frac{{\delta ^{ - \gamma }k}}{{(N - k)^2}}\frac{{{\mathrm{e}}^{\frac{{ - \left( {\frac{g}{N} - \mu } \right)^2}}{{2\sigma ^2}}}}}{{N\sqrt {2{\mathrm{\pi }}\sigma ^2} }}} \hfill \\ {} \hfill & {} \hfill & { \times \left( {1 - \frac{{g\varepsilon }}{{v_{\mathrm{c}}}}} \right)^{\beta - 1}\left( {\frac{{g\varepsilon }}{{v_{\mathrm{c}}}}} \right)^{\alpha}v_{\mathrm{c}}^{ - 1}dg,} \hfill \end{array}{}$$

where *ε* = ½*v*^2^(1 − *a*_0_)*τ*_t_(*K*_A_ − *K*_I_)$\nabla$*C*[(*K*_A_ + *C*)(*K*_I_ + *C*)]^−1^ groups all the constant terms of the expression for the chemotactic velocity in Eq. (3).

The relative performance of phenotypes as a function of junction number *n* for both the log-sensing and linear-sensing regimes can be calculated as (Fig. 5d)

13 $${f_{g,{T}_{\mathrm{B}}}}{P}_{n} =	 \mathop{\sum}\limits_{k = 1}^{\infty} \mathop{\sum}\limits_{N = 1}^k {\frac{{\lambda} ^k{\mathrm{e}}^{ {-} {\lambda} }}{k!}{\mathrm{e}}^{ \frac{-N}{{\delta (k - N)}}}\left( {\frac{N}{{k - N}}} \right)^{{\gamma} - 1}} \\ 	{ \times \frac{{\delta} ^{ - {\gamma} }k}{{(N - k)^2}}\frac{{{\mathrm{e}}^{\frac{{ - \left( {\frac{g}{N} - \mu } \right)^2}}{{2{\sigma} ^2}}}}}{{N\sqrt {2{{\pi }}{\sigma} ^2} }}} \\ 	{ \times \frac{{{\mathrm{\Gamma }}(\alpha + \beta )}}{{{{\Gamma }}(\alpha ){\kern 1pt} {{\Gamma }}(\beta )}}(1 - T_{\mathrm{B}})^{{\beta} - 1}{T}_{\mathrm{B}}^{{\alpha} - 1}} \\ 	{ \times B_0(4{{\pi }}Dt)^{ - 1/2}{\mathrm{e}}^{ \frac{{-d_n^2}}{{4Dt}}}{\mathrm{e}}^{ -\mathop {\sum}\nolimits_{i = 1}^n \frac{{ {iy_{\mathrm{v}}v_{{\mathrm{c}}_i}} }}{{4D}}}}.$$

The number of bacteria *N*_B_ reaching each junction *n* in the T-maze for a population expressing variation in the phenotypes *g*, *T*_B_ is

14 $$N_{\mathrm{B}}(n,t) = \mathop {\smallint }\limits_0^1 dT\mathop {\smallint }\limits_0^\infty dg\;f_{g,T_{\mathrm{B}}}P_n{.}$$

The heterogeneous sorting index is given by the ratio

15 $$H_n = N_{\mathrm{B}}(n,t)/\left[B_n\left( {\overline {T}_{\mathrm{B}} } \right)S_n\left( {\bar g,\overline {T}_{\mathrm{B}} } \right)\right]{,}$$

where the denominator is the number of bacteria expected at junction *n* from a population with mean values $\overline {T}_{\mathrm{B}}$ = *α/(α* *+* *β*)= 0.22, and $\bar g$ = 76, extracted from the initial distributions ${\cal{B}}$(*T*_B_;*α*,*β*) and *h*_*g*_, respectively. Since *N*_B_ is a function of time, the index was evaluated at the experimental time poin*t*s *t*_*i*_. The heterogeneous sorting index at each junction from the experiments (Fig. 5c) was used to fit the only free parameter of the model, *σ* = 7.8.

The marginal distributions at each junction of the tumble bias and pathway gain as a function of time are

16 $$b_{T_{\mathrm{B}}}(n,t) = \mathop {\smallint }\limits_0^\infty dg\;f_{g,T_{\mathrm{B}}}P_n = {\cal{B}}\left( {T_{\mathrm{B}};\alpha \left( {n,t} \right),\beta \left( {n,t} \right)} \right){,}$$

and

17 $$h_g(n,t) = \mathop {\smallint }\limits_0^1 dT\;f_{g,T_{\mathrm{B}}}P_{n}{,}$$

where *α*(*n*, *t*), *β*(*n*, *t*) are the fitted sets of parameters of the marginal distribution $b_{T_{\mathrm{B}}}(n,t)$ calculated at junction *n*. We use Eqs. (16) and (17) to calculate the distribution of the chemotactic velocity (Fig. 5b) at each junction as a function of time

18 $$\begin{array}{*{20}{l}} {d_{v_{\mathrm{c}}}(n,t)} \hfill & = \hfill & {\frac{{{\mathrm{\Gamma }}\left( {\alpha \left( {n,t} \right) + \beta \left( {n,t} \right)} \right)}}{{{\mathrm{\Gamma }}\left( {\alpha (n,t)} \right.{\mathrm{\Gamma }}\left( {\beta \left( {n,t} \right)} \right)}}\mathop {\int}\limits_0^\infty {h_g\left( {n,t} \right)} } \hfill \\ {} \hfill & {} \hfill & { \times \left( {1 - \frac{{g\varepsilon }}{{v_{\mathrm{c}}}}} \right)^{\beta (n,t) - 1}\left( {\frac{{g\varepsilon }}{{v_{\mathrm{c}}}}} \right)^{\alpha (n,t)}v_{\mathrm{c}}^{ - 1}dg.} \hfill \end{array}{.}$$

### Reporting Summary

Further information on experimental design is available in the Nature Research Reporting Summary linked to this article.

## Supplementary information

Supplementary Information

Reporting Summary

Source Data

## Data Availability

The data that support the findings of this study are available from the corresponding author upon request.
