# Supplementary material for: Bacterial chemotaxis in a microfluidic T-maze reveals strong phenotypic heterogeneity in chemotactic sensitivity
Source: Nat Commun. 2019 Apr 23;10:1877. doi: 10.1038/s41467-019-09521-2 (PMC6478840; doi:10.1038/s41467-019-09521-2)
Supplement: Supplementary file 1 — Supplementary Information [file 41467_2019_9521_MOESM1_ESM.pdf]

Supplementary Information for

Bacterial chemotaxis in a microfluidic T-maze reveals strong phenotypic heterogeneity in chemotactic sensitivity

M. Mehdi Salek*, Francesco Carrara*, Vicente Fernandez, Jeffrey S. Guasto, and Roman Stocker

Corresponding author:
romanstocker@ethz.ch

Supplementary Discussion

The effects of swimming speed, adaptation time, tumble bias, and pathway gain on the accumulation length of bacterial concentration profiles.

A monoclonal population of bacteria naturally expresses a spectrum of phenotypic traits contributing to chemotactic performance, namely swimming speed, tumble bias, pathway gain and adaptation time. In general, one expects this spectrum of values to be reflected in a distribution of the chemotactic sensitivity coefficient χ_0 and the diffusivity D. Our conclusion that cells are sorted in the T-maze based primarily on chemotactic sensitivity coefficient rests on two main results: 1) the increase in the slope, the inverse of the accumulation length $L_c = D/v_c$, of the bacterial concentration profiles in the linear-sensing regime (and the absence of a decrease in the log-sensing regime), the consequences of which for the chemotactic sensitivity coefficient will be discussed below; and 2) the increased number of cells reaching the last junction relative to the number of cells expected based on a model with a constant chemotactic sensitivity coefficient (which is discussed in the main text). Here we discuss the effects on the bacterial concentration profiles of the four phenotypic traits of *E. coli* that have been considered in previous studies[1,2], namely the swimming speed, the adaptation time, the tumble bias, and the pathway gain. To see that the increase in the slope of the bacterial concentration profile detected in Supplementary Fig. 1 (Main Text Equation 7) is mainly due to an increase in the pathway gain (and not to variation in swimming speed, adaptation time, or tumble bias), consider the following equations for the chemotactic velocity and the chemotactic sensitivity coefficient (following the derivation provided in ref. 3):

$$v_c = \chi_0\,(K_A - K_I)[(K_I + C)(K_A + C)]^{-1}\,\nabla C, \qquad (1)$$

$$\chi_0 = v^2\, NH\,(1 - a_0)\,/\,[N\alpha(k_R + k_B)a_0(1 - a_0) + 2\tau_0^{-1}], \qquad (2)$$

where v is the swimming speed, v_c is the chemotactic velocity, χ_0 is the chemotactic sensitivity coefficient, C is the chemoattractant (methylaspartate) concentration, K_I and K_A are the dissociation constants for inactive and active Tar receptors, a_0 denotes the steady-state kinase activity, H is the motor amplification coefficient, τ_0 is the average run time, N is the number of receptors per cluster, k_R and k_B are relative rates of receptor methylation and demethylation, and α is the gain term in the free energy due to receptor methylation.

i) We first note that in the equation for the accumulation length $L_c = D/v_c$, the quadratic dependence on the swimming speed in the diffusivity term (Main Text Equation 6) cancels out with the quadratic dependence on the swimming speed in the chemotactic sensitivity coefficient (Supplementary Equation 2), and hence in the chemotactic velocity term (Supplementary Equation 1). As a result, there is no dependence of the accumulation length on the swimming speed (Main Text Equation 7).

(ii) To understand the effect of the adaptation time, we note that in the denominator of Supplementary Equation 2, the first term, $N\alpha(k_R + k_B)a_0(1 - a_0)$, is related to the adaptation timescale for the methylation dynamics. Consider the first-order Taylor expansion of the methylation dynamics at the adapted state, $d(m–m_0)/dt = F'(a_0)\,(a–a_0)$, where $(a–a_0) = \partial a/\partial m_{|m=m_0}\,(m–m_0)$ and $\partial a/\partial m_{|m=m_0} = \alpha N a_0(1 - a_0)$ from Supplementary Equation 7 below (for a derivation, see the section "*Chemotaxis pathway in E. coli: receptor cooperativity, motor gain, and tumble bias*" below). By taking the temporal derivative, we obtain

$$d(a - a_0)/dt = \alpha N a_0(1 - a_0)\, d(m - m_0)/dt = \alpha N a_0(1 - a_0)F'(a_0)\,(a - a_0), \qquad (3)$$

where we can identify the adaptation timescale for the kinase activity

$$\tau_m = - \alpha N a_0\,(1 - a_0)\,F'(a_0) = \alpha N a_0(1 - a_0)\,(k_R + k_B)$$

by substituting $F'(a_0)$ from Supplementary Equation 8. The chemotactic sensitivity coefficient in Supplementary Equation 2 thus becomes

$$\chi_0 = v^2\, NH\,(1 - a_0) / [\tau_m^{-1} + 2\tau_0^{-1}]. \qquad (4)$$

For typical values of the constants $N < 10$, $\alpha = 2$ (ref. 4), $\{k_R, k_B\} \sim 10^{-2}\,s^{-1}$ and $a_0 = 0.33$, we find the adaptation time τ_m appearing in Supplementary Equation 4 to be much greater than 1 s, specifically $\tau_m = 28$ s if we take the cluster size as $N = 6$ (ref. 5). Conversely, the second term in the square brackets of Supplementary Equation 4 is of order 1 s, since the run time of the cells is $\tau_0 \sim 1$ s. The first term of the denominator in Supplementary Equation 4 is thus much smaller than the second. As such, variation in the adaptation time makes little contribution to variation in the chemotactic velocity, and for this reason we neglected the dependence of the chemotactic sensitivity coefficient on the adaptation time. By neglecting the small contribution of the adaptation time, the chemotactic sensitivity coefficient can be approximated as

$$\chi_0 \sim ½\, v^2\, NH\,(1 - a_0)\,\tau_0 = ½\, v^2\, g\,(1 - a_0)\,\tau_t / T_B \qquad (5)$$

after having expressed the run time in Supplementary Equation 4 as $\tau_0 = \tau_t / T_B$, where τ_t is the tumble time and T_B is the tumble bias, and the pathway gain, $g = NH$, determined by the number of receptors N and the motor gain H.

(iii) The lack of dependence on the tumble bias, T_B, of the accumulation length $L_c = D/v_c$ in the bacterial concentration profile (Main Text Equation 7) can be understood by considering the run time, τ_0, where $\tau_0 = \tau_t / T_B$. By combining Supplementary Equation 1 with Supplementary Equation 5:

$$L_c = 2/3\, v^2\, \tau_0\, (1 - \langle\cos\theta\rangle)^{-1} / \{(K_A - K_I)\nabla C(x)[(K_A + C(x))\,(K_I + C(x))]^{-1}\, g\, (1 - a_0)\, v^2 \tau_0 \}, \quad (6)$$

the run time τ_0 cancels out in the chemotactic accumulation length. A change in the run duration (i.e., a change in the tumble bias) thus does not affect the bacterial concentration profile.

In summary, ruling out the other traits that were potential candidates for the observed decrease in the bacterial accumulation length with distance into the T-maze supports our conclusion that the main factor responsible for the observed heterogeneity is heterogeneity in the pathway gain, $g = NH$, determined by the number of receptors N and the motor gain H.

Chemotaxis pathway in *E. coli*: receptor cooperativity, motor gain, and tumble bias

The methylation dynamics catalyzed by CheR and CheB are captured by the enzymatic reaction model[6]

$$dm/dt = F(a) = V_R\,(1 - a)/[K_R + (1 - a)] - V_B\, a/(K_B + a), \quad (7)$$

where m is the methylation state of the receptor clusters, $a = T^* / T_{tot}$ is the kinase activity (bounded between 0 and 1), T^* is the number of active receptors and T_{tot} is the total number of receptors. Following Shimizu *et al.*[6], the kinase activity here is assumed to be in the range $a < 3/4$. $K_R = 0.32$ and $K_B = 0.30$ are the Michaelis-Menten constants for the methylation and demethylation reactions, while $V_R = 0.010\ s^{-1}$ and $V_B = 0.013\ s^{-1}$ are the rates of the methylation

and demethylation reactions[6], respectively (all concentrations are expressed in units of the CheA kinase concentration). This nonlinear model can be linearized[7,8] around the adapted state a_0 as

$$dm/dt = k_R (1 - a) - k_B a \, , \qquad (8)$$

where k_R and k_B are rescaled rates that are assumed constant in our model. These two constants can be derived by evaluating Supplementary Equation 7 at equilibrium to determine $a_0 = 0.33$ and by calculating the derivative evaluated at $a_0 = 0.33$ of Supplementary Equation 7, $F'(0.33) = -0.013$. This gives the values $k_R = -a_0\, m'(a_0) = 0.43 \times 10^{-2}\ s^{-1}$ and $k_B = -m'(a_0) - k_R = 0.87 \times 10^{-2}\ s^{-1}$. For the linear model, the adapted kinase activity gives at equilibrium

$$a_0 = k_R / (k_B + k_R), \qquad (9)$$

which is independent of the chemoattractant concentration, therefore allowing perfect adaptation. The kinase activity a can be measured in FRET (fluorescence resonance energy transfer) experiments by assuming a fast phosphotransfer. A linear relationship between the concentration of phosphorylated CheY and a is found, CheY-P = $\beta\, a$ (ref. 6). The adapted kinase activity was recently measured in FRET experiments at the single-cell level and was found to vary in the range $a_0 = 0.302 \pm 0.071$ (ref. 9), which is consistent with the value of 0.33 that we used, derived from earlier population-scale measurements[6].

According to the Monod-Wyman-Changeux (MWC) model[10] the receptors are organized in clusters of size N, which are strongly coupled and switch off and on together. The kinase activity a is a function of the free energy differences (in units of thermal energy kT) associated with the methylation $f(m)$ and ligand $f(C)$, which is amplified by the cooperativity N

$$a = 1 / (1 + [f(m)\, f(C)]^N), \qquad (10)$$

where $f(m) = e^{\alpha(m_0 - m)}$ with α the free-energy change per added methyl group, and $f(C) = (C + K_I)/(C + K_A)$ with $K_I = 18$ μM and $K_A = 2.9$ mM being the inactivation and activation constants of the methylaspartate receptors[4,6].

The kinase activity a modulates the motor switching between counterclockwise and clockwise rotation and thus determines the tumbling rate λ, as

$$\lambda(a_0,C) = \lambda_0\,(a/a_0)^H + 2D_r\,, \qquad (11)$$

where λ_0 is the adapted tumbling rate, H is the motor gain and $D_r = 0.063$ s^{-1} is the rotational diffusivity of the cell[8]. Thus, by regulating the tumbling rate, the kinase activity determines the tumble bias of the cell[11]. Considering τ_t the tumbling time and τ_r the run time, the tumble bias T_B is defined as

$$T_B = \tau_t \,/\, (\tau_t + \tau_r) = [1 + (\lambda_0(a/a_0)^H + 2D_r)^{-1} / \,\tau_t]^{-1}\,. \qquad (12)$$

In accordance with the literature[7,8], here we consider variation in the tumble bias due to variability in the run time rather than the tumbling time.

Supplementary references

1. Frankel, N. W. *et al.* Adaptability of non-genetic diversity in bacterial chemotaxis. e*life* **3**:e03526 (2014).
2. Waite, A. J., Frankel, N. W., Dufour, Y. S., Johnston, J. & Emonet, T. Non-genetic diversity modulates population performance. *Mol Sys Bio* **12,** 1–14 (2016).
3. Menolascina, F. *et al.* Logarithmic sensing in *Bacillus subtilis* aerotaxis. *Syst. Biol. Appl.* **3,** 1–8 (2017).
4. Lazova, M. D., Ahmed, T., Bellomo, D., Stocker, R. & Shimizu, T. S. Response rescaling in bacterial chemotaxis. *Proc. Natl. Acad. Sci. U. S. A.* **108,** 13870–13875 (2011).
5. Mello, B. a & Tu, Y. Effects of adaptation in maintaining high sensitivity over a wide range of backgrounds for *Escherichia coli* chemotaxis. *Biophys. J.* **92,** 2329–37 (2007).

6. Shimizu, T. S., Tu, Y. & Berg, H. C. A modular gradient-sensing network for chemotaxis in *Escherichia coli* revealed by responses to time-varying stimuli. *Mol. Syst. Biol.* **6,** 1–14 (2010).

7. Jiang, L., Ouyang, Q. & Tu, Y. Quantitative modeling of *Escherichia coli* chemotactic motion in environments varying in space and time. *PLoS Comput. Biol.* **6,** (2010).

8. Si, G., Wu, T., Qi, O. & Tu, Y. Pathway-based mean-field model for *Escherichia coli* chemotaxis. *Phys. Rev. Lett.* **109,** 0–4 (2012).

9. Keegstra, J. *et al.* Phenotypic diversity and temporal variability in a bacterial signaling network revealed by single-cell FRET. e*life* **6**:e27455 (2017).

10. Monod, J., Wyman, J. & Changeux, J. P. On the nature of allosteric transitions: A plausible model. *J. Mol. Biol.* **12,** 88–118 (1965).

11. Cluzel, P., Surette, M. & Leibler, S. An ultrasensitive bacterial motor revealed by monitoring signaling proteins in single cells. *Science* **287,** 1652–1655 (2000).

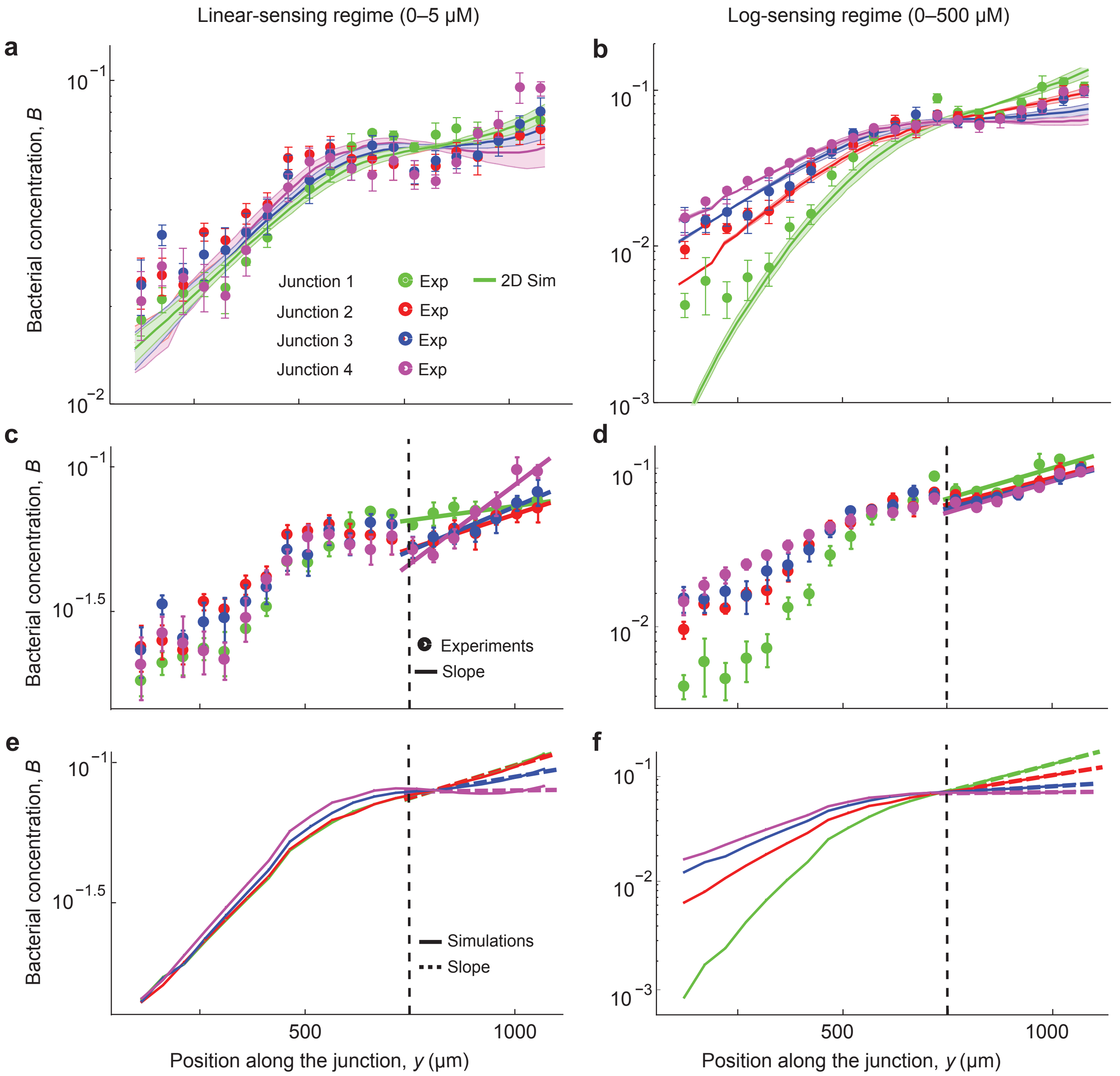

Supplementary Figure 1 | *E. coli* accumulation profiles in the T-maze. Panels show the results for the linear-sensing (0–5 μM MeAsp, **a, c, e**), and log-sensing regimes (0–500 μM MeAsp, **b, d, f**). (**a**, **b**) Average relative bacterial concentrations for the experiments (circles) and for the 2-D numerical simulations (lines; with the assumption of a chemotactically homogeneous population). The slopes of the accumulation profiles were calculated on the section of the T-junction between 750 and 1100 μm (on the source side of the channel, to the right of the dashed vertical line in the plots; Methods) for the experimental results (**c**, **d**) and the simulations (**e**, **f**). Numerical values with associated standard errors are provided in Supplementary Table 1.

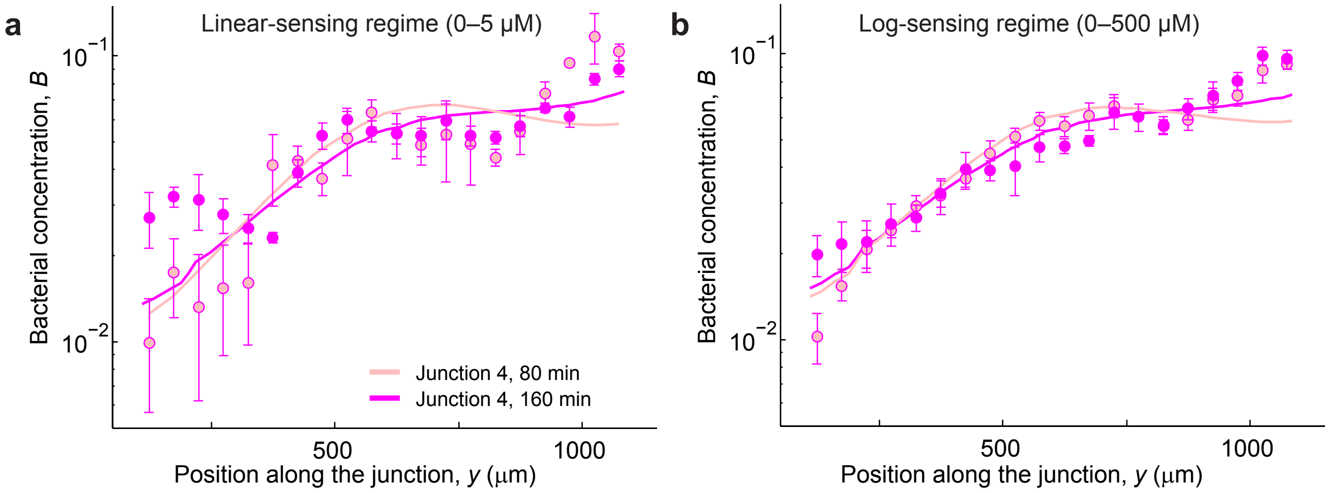

Supplementary Figure 2 | Comparison between the experimental *E. coli* accumulation profiles in junction 4 (circles) and the 2-D simulations (lines) at two time points. (**a**) linear-sensing (0–5 μM MeAsp), and (**b**) log-sensing (0–500 μM MeAsp) regimes. Error bars for the experimental data are ± s.d. over the three experimental replicates.

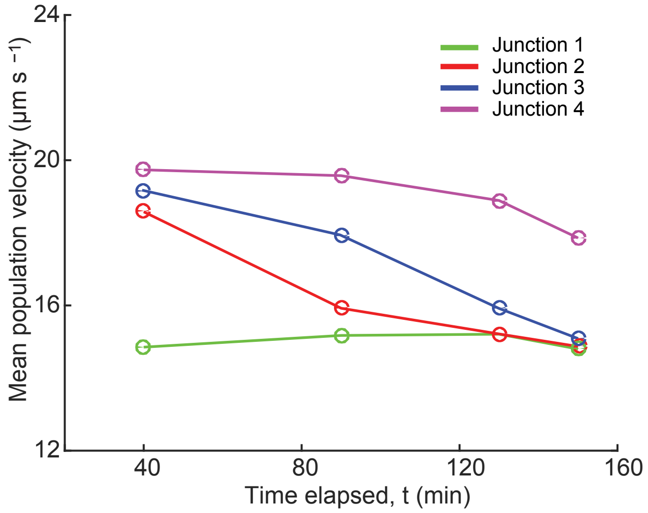

Supplementary Figure 3 | The mean swimming speed of the population increases at consecutive junctions and declines through time.

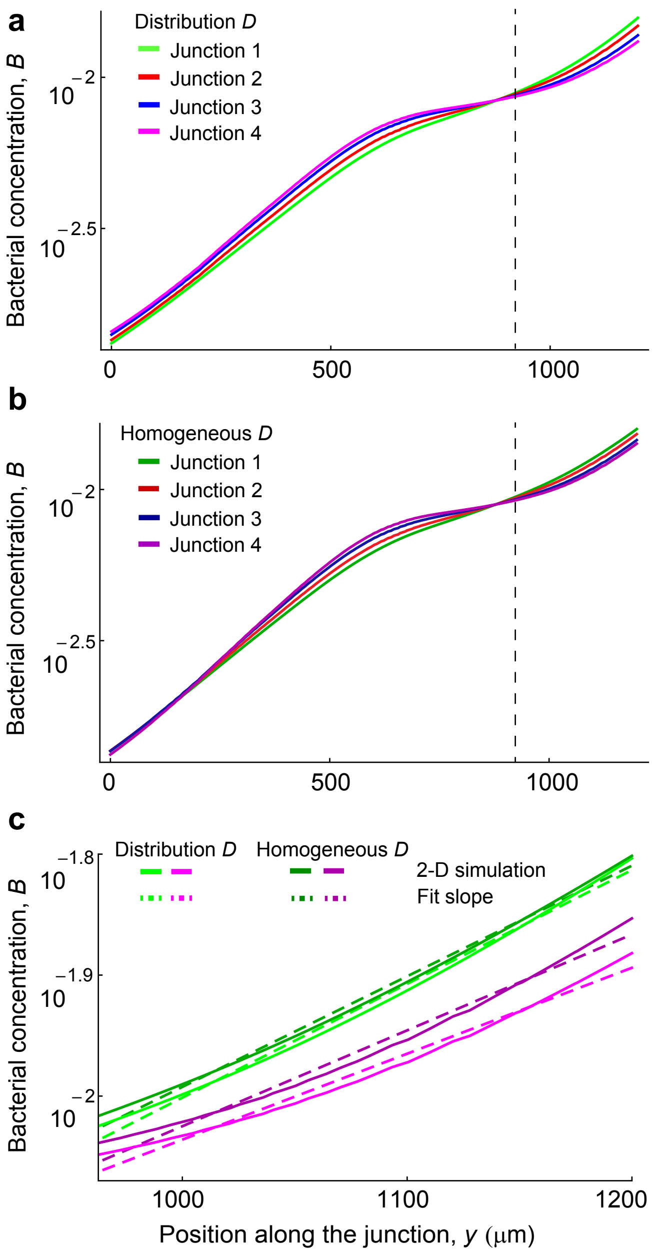

Supplementary Figure 4 | Bacterial accumulation profiles in the T-maze for the linear-sensing regime (0–5 μM MeAsp). (**a**) Relative bacterial concentrations along each junction for the numerical 2-D simulations under the assumption of a chemotactically homogeneous population, with a heterogeneous diffusion coefficient characterized by a Gaussian distribution with mean D = 330 μm^2 s^{-1} and standard deviation 100 μm^2 s^{-1}. (**b**) Relative concentrations for the numerical 2-D simulations under the assumption of a chemotactically homogeneous population, with a homogeneous diffusion coefficient D = 330 μm^2 s^{-1}. (**c**) Accumulation profiles for the two populations at junctions 1 (green) and 4 (magenta), and slopes calculated on the section of the T-junction between 950 and 1200 μm (on the source side of the channel, to the right of the dotted vertical line in panels **a**,**b**). Note that the slope at junction 4 for a population presenting a distribution of diffusivities is lower than that for the population with a homogeneous value for the diffusion with the same mean. Numerical values for all junctions with associated standard errors are provided in Supplementary Table 2.

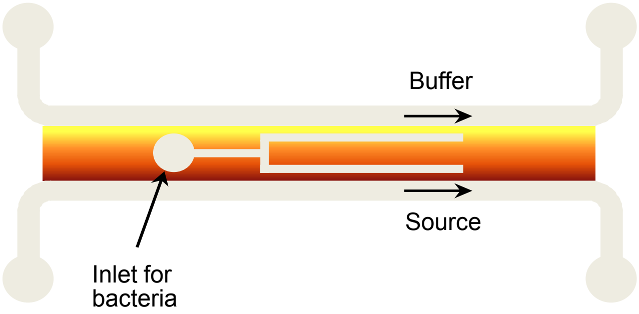

Supplementary Figure 5 | Schematic of the single-junction device. In this device, the gradient generated between the source and sink (buffer) corresponds to that in the last junction (junction 4) of the T-maze device. Comparison between the performance of bacteria from an unsorted population within this device and that of bacteria in junction 4 of the T-maze provides a test of sorting on the basis of chemotactic performance within the maze. The distance between the source and buffer is 1800 µm. The section of the junction that lies parallel to the gradient measures 1200 µm. The junction runs from a point 300 µm from the sink to 1500 µm from the sink. The levels of chemoattractant in the single-junction device were chosen so that the concentration profile matches that at junction 4 of the T-maze. The level of the chemoattractant for the sink and the source were set to 2.34 µM and 5.15 µM for the experiments in the linear-sensing regime and 234 µM and 515 µM for the experiments in the log-sensing regime, corresponding to the level of chemoattractant increasing within the junction from 2.81 µM to 4.68 µM for the experiments in the linear-sensing regime and 281 µM to 468 µM for the experiments in the log-sensing regime. The concentration profiles along the junctions thus match in the two devices.

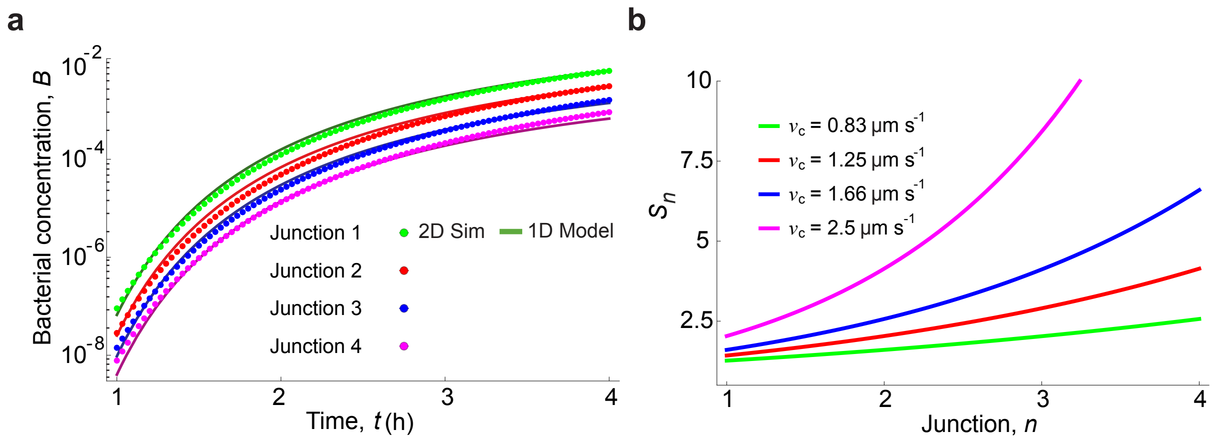

Supplementary Figure 6 | Sorting within the T-maze. (**a**) Accumulation of bacteria in the four junctions as a function of time, showing the correspondence between the 2-D simulations (circles) and the 1-D model (lines). (**b**) Sorting index, S_n (v_c / D), as a function of the number of the junction, for different chemotactic velocities.

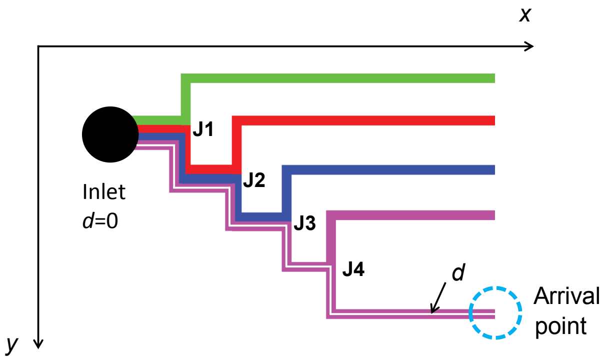

Supplementary Figure 7 | Schematic of the path travelled by cells through different junctions. In the analytical approximation of the partial differential equations (1-D model), the solution in each branch is independent from the others. The linear distance travelled by the cells in each branch, shown by *d* (white line), is simply the length of the associated colour-coded path.

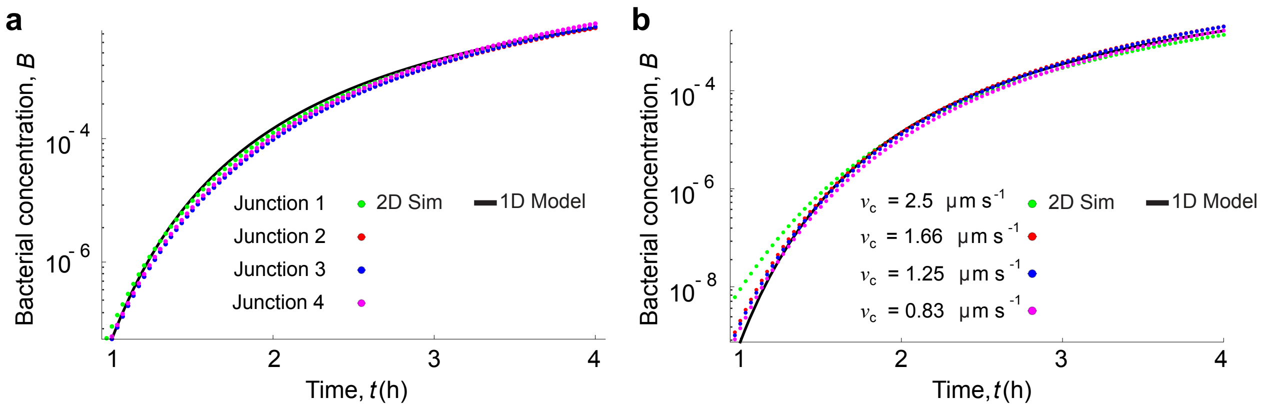

Supplementary Figure 8 | Collapse of the bacterial accumulations in the T-maze as a function of time. (**a**) Results from the 2-D simulations and the 1-D diffusion model calculated at the four junctions. (**b**) Results from the 1-D advection–diffusion model for four different velocities.

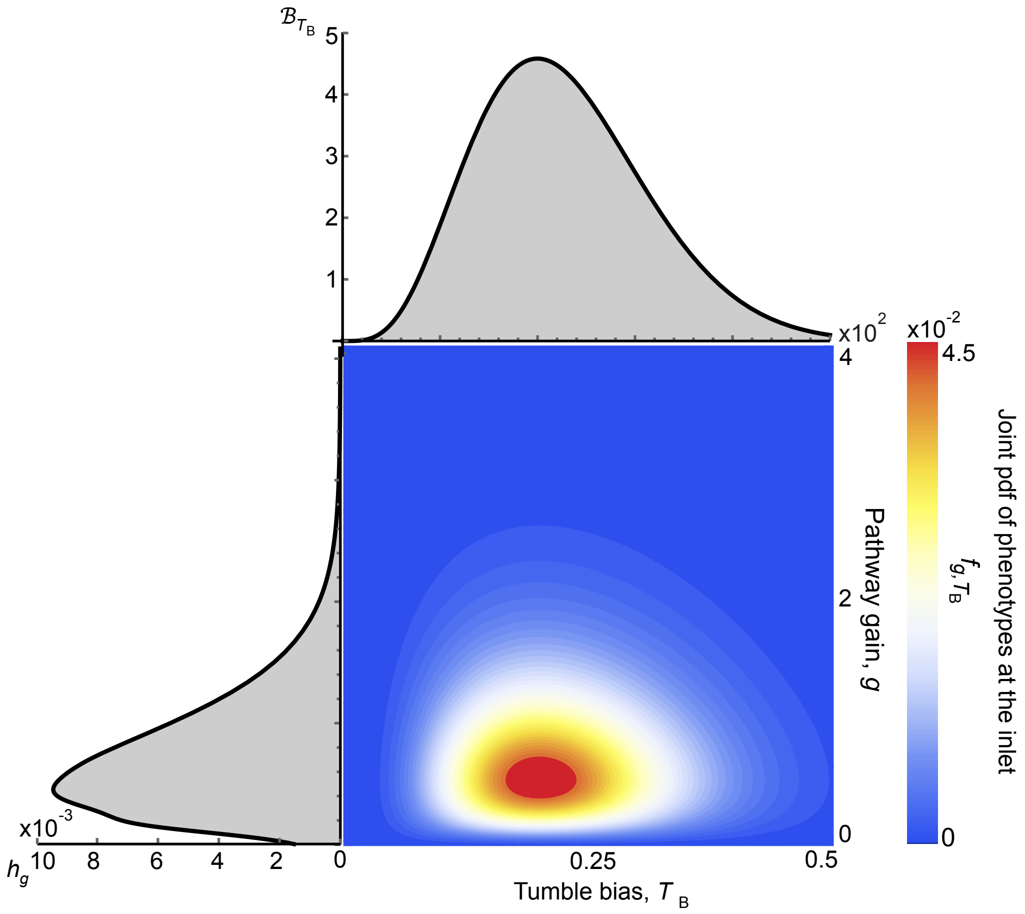

Supplementary Figure 9 | Joint probability density function of phenotypes as a function of pathway gain and tumble bias for a monoclonal population of cells at the inlet. The gain, g, is extracted from the distribution h_g (Eq. 10, σ = 7.8), and the tumble bias T_B from a Beta distribution $\mathcal{B}(T_B;\alpha,\beta)$ with parameters α = 5 and β = 17. Grey curves show h_g (left) and T_B (top). Colour scale indicates the values taken by the joint probability density function.

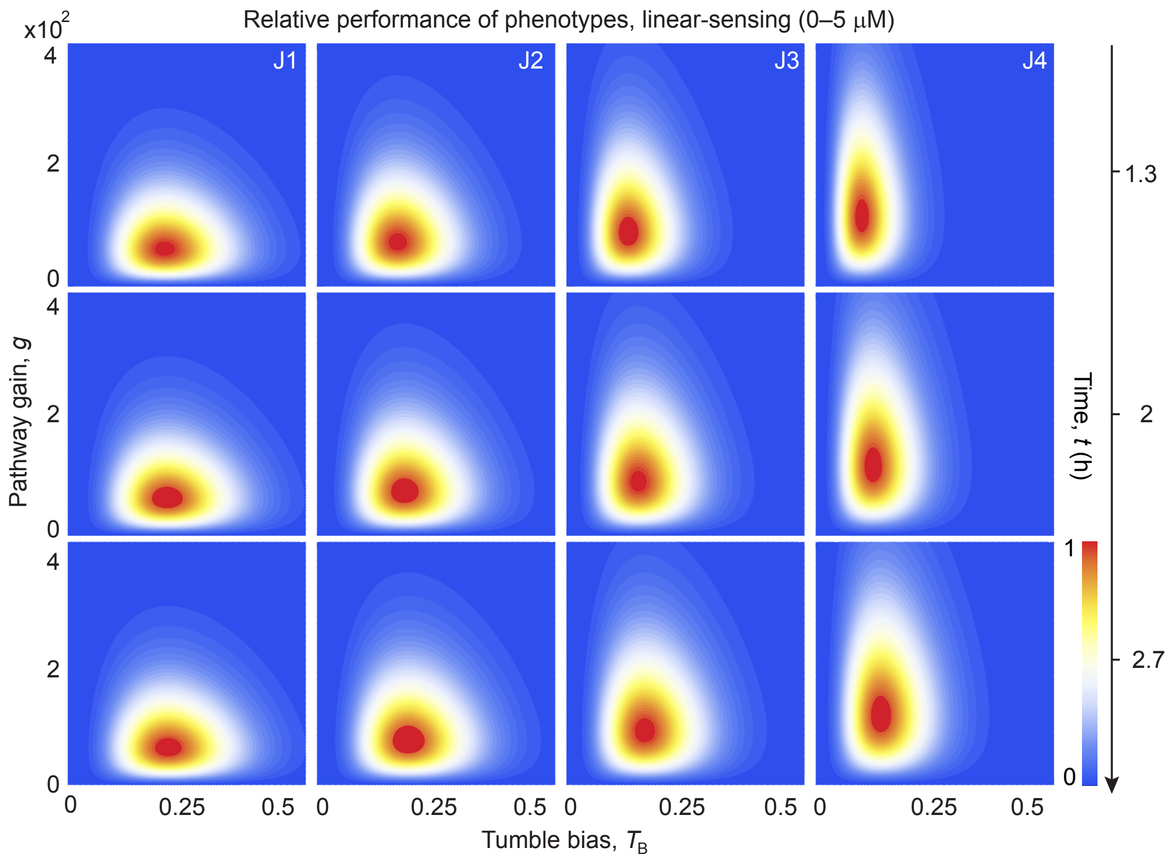

Supplementary Figure 10 | Relative performance of phenotypes as a function of pathway gain and tumble bias for a monoclonal population of cells reaching each junction in the T-maze and calculated at three different time points for the linear-sensing regime.

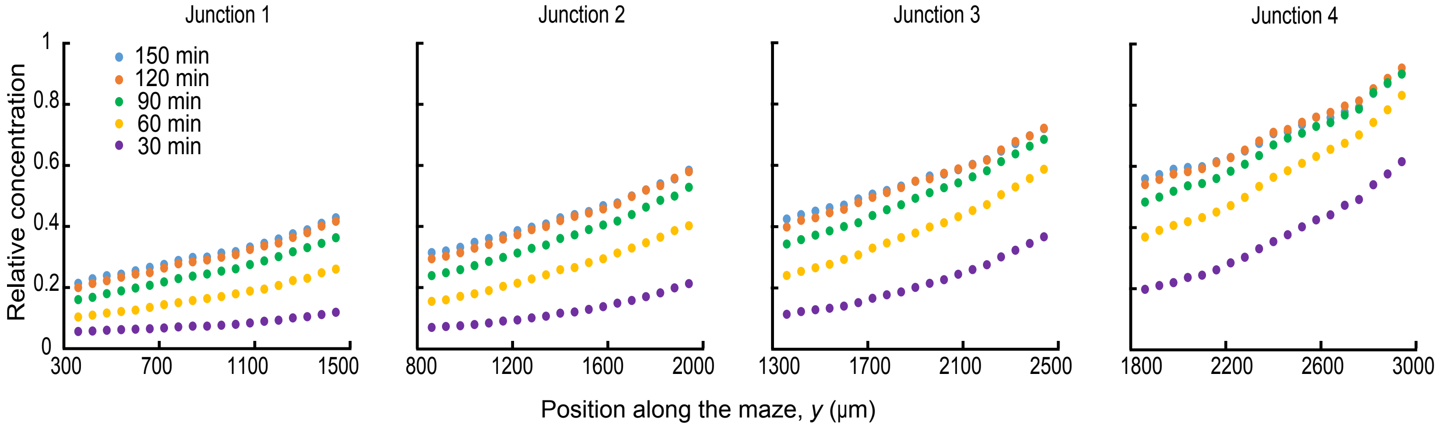

Supplementary Figure 11 | Gradient development through time in each junction of the maze. The *x* axes show the location of each junction along the maze with respect to the sink (for example, junction 1 runs between points located 300 µM to 1500 µM from the sink).

Supplementary Table 1 | Slopes of the bacterial concentration profiles in the simulations and experiments with *E. coli*, for the linear-sensing regime (0–5 μM) and for the log-sensing regime (0–500 μM).

	Slope (μm^{-1}) for linear sensing		Slope (μm^{-1}) for log-sensing	
**Junction**	**Experiments**	**Simulations**	**Experiments**	**Simulations**
1	$4.5 \pm 1.5 \times 10^{-4}$	$8.8 \pm 0.4 \times 10^{-4}$	$1.7 \pm 0.4 \times 10^{-3}$	$2.2 \pm 0.1 \times 10^{-3}$
2	$1.1 \pm 0.1 \times 10^{-3}$	$8.4 \pm 0.4 \times 10^{-4}$	$1.5 \pm 0.3 \times 10^{-3}$	$1.4 \pm 0.1 \times 10^{-3}$
3	$1.4 \pm 0.2 \times 10^{-3}$	$4.8 \pm 0.5 \times 10^{-4}$	$1.6 \pm 0.3 \times 10^{-3}$	$0.5 \pm 0.1 \times 10^{-3}$
4	$2.5 \pm 0.3 \times 10^{-3}$	$3.5 \pm 0.7 \times 10^{-5}$	$1.8 \pm 0.3 \times 10^{-3}$	$0.8 \pm 0.4 \times 10^{-4}$

Supplementary Table 2 | Slopes of the bacterial concentration profiles along each junction in the simulations for heterogeneous and homogeneous populations. The heterogeneous population has a Gaussian distribution of diffusion coefficients (mean D = 330 μm^2 s^{-1} and standard deviation 100 μm^2 s^{-1}) and the homogeneous population has a diffusion coefficient of D = 330 μm^2 s^{-1}, for the linear-sensing regime (0–5 μM).

	Slope (μm^{-1})	
**Junction**	**Distribution *D***	**Homogeneous D**
1	$9.4 \pm 0.1 \times 10^{-4}$	$9.1 \pm 0.1 \times 10^{-4}$
2	$8.6 \pm 0.1 \times 10^{-4}$	$8.7 \pm 0.1 \times 10^{-4}$
3	$7.7 \pm 0.2 \times 10^{-4}$	$8.3 \pm 0.2 \times 10^{-4}$
4	$7.1 \pm 0.2 \times 10^{-4}$	$7.9 \pm 0.2 \times 10^{-4}$
