# Supplementary material for: Bacterial chemotaxis in a microfluidic T-maze reveals strong phenotypic heterogeneity in chemotactic sensitivity
Source: Nat Commun. 2019 Apr 23;10:1877. doi: 10.1038/s41467-019-09521-2 (PMC6478840; doi:10.1038/s41467-019-09521-2)
Supplement: Supplementary file 2 — Reporting Summary [file 41467_2019_9521_MOESM2_ESM.pdf]

nature research

Corresponding author(s): Roman Stocker

Last updated by author(s): Jan 11, 2019

Reporting Summary

Nature Research wishes to improve the reproducibility of the work that we publish. This form provides structure for consistency and transparency in reporting. For further information on Nature Research policies, see Authors & Referees and the Editorial Policy Checklist.

Statistics

For all statistical analyses, confirm that the following items are present in the figure legend, table legend, main text, or Methods section.

n/a	Confirmed	
☐	☒	The exact sample size (n) for each experimental group/condition, given as a discrete number and unit of measurement
☐	☒	A statement on whether measurements were taken from distinct samples or whether the same sample was measured repeatedly
☐	☒	The statistical test(s) used AND whether they are one- or two-sided<br>*Only common tests should be described solely by name; describe more complex techniques in the Methods section.*
☒	☐	A description of all covariates tested
☒	☐	A description of any assumptions or corrections, such as tests of normality and adjustment for multiple comparisons
☐	☒	A full description of the statistical parameters including central tendency (e.g. means) or other basic estimates (e.g. regression coefficient) AND variation (e.g. standard deviation) or associated estimates of uncertainty (e.g. confidence intervals)
☐	☒	For null hypothesis testing, the test statistic (e.g. F, t, r) with confidence intervals, effect sizes, degrees of freedom and P value noted<br>*Give P values as exact values whenever suitable.*
☒	☐	For Bayesian analysis, information on the choice of priors and Markov chain Monte Carlo settings
☒	☐	For hierarchical and complex designs, identification of the appropriate level for tests and full reporting of outcomes
☒	☐	Estimates of effect sizes (e.g. Cohen's d, Pearson's r), indicating how they were calculated

Our web collection on statistics for biologists contains articles on many of the points above.

Software and code

Policy information about availability of computer code

Data collection	NIS-Elements was used for microscopic data collection.
Data analysis	Matlab, Mathematica and Comsol were used for simulations and data analysis.

For manuscripts utilizing custom algorithms or software that are central to the research but not yet described in published literature, software must be made available to editors/reviewers. We strongly encourage code deposition in a community repository (e.g. GitHub). See the Nature Research guidelines for submitting code & software for further information.

Data

Policy information about availability of data

All manuscripts must include a data availability statement. This statement should provide the following information, where applicable:

- Accession codes, unique identifiers, or web links for publicly available datasets
- A list of figures that have associated raw data
- A description of any restrictions on data availability

Provide your data availability statement here.

Field-specific reporting

Please select the one below that is the best fit for your research. If you are not sure, read the appropriate sections before making your selection.

☐ Life sciences ☐ Behavioural & social sciences ☒ Ecological, evolutionary & environmental sciences

For a reference copy of the document with all sections, see nature.com/documents/nr-reporting-summary-flat.pdf

Ecological, evolutionary & environmental sciences study design

All studies must disclose on these points even when the disclosure is negative.

Study description	This study quantitatively addressed the amount of heterogeneity in a monoclonal population of bacterial cells by employing a microfluidic T-maze device. Unless otherwise indicated three replicates were generated to obtain results with sufficient accuracy for the study.
Research sample	For this study, we used E. coli HCB 33 (also known as RP437) and two Marinobacter adhaerens strains, a wild-type strain (WT) labeled with YFP and a chemotaxis-deficient mutant that constitutively expressed cyan fluorescent protein (ΔcheA-cfp).
Sampling strategy	For each experiment a single colony was picked from a culture plate to inoculate. Cells were picked at mid-exponential phase at the same OD to make sure equal number of cells were used across different replicates. Bacteria were washed and diluted and they were injected into the microfluidic device. Bacteria were imaged at the mid-plane of the microfluidic device using phase contrast microscopy. At regular time intervals, each junction in the maze was imaged and bacterial trajectories were extracted.
Data collection	We used video microscopy to capture the motion of bacteria as they migrate up or down the gradient at each subsequent junction through the maze and cells were tracked using image analysis routines based on intensity thresholding in custom, automated routines in MATLAB (The MathWorks).
Timing and spatial scale	The microfluidic consists of three parallel sections, with two parallel channels for the chemoattractant source and buffer and the maze test section located between them. Source and buffer are located 3200 μM from each other. Junction 1, 2, 3, and 4 are located 300 μM to 1500 μM, 800 μM to 2000 μM, 1300 μM to 2500 μM, and 1800 μM to 3000 μM from the sink respectively. In all replicated, bacteria were imaged at the mid-plane of the maze test section in the microfluidic device using phase contrast microscopy (10× objective; Andor Zyla camera with 6.5 μm/pixel; N.A. = 0.30; with additional magnification 1.5×) at 25 frames/s. The chemoattractant gradient was allowed to develop through the device over 2.5–3 h in the absence of bacteria to allow time for diffusion from the source to the buffer channel. This time was sufficient to allow the concentration field to become established in the device. Then bacteria were injected in the device and were imaged up to three hours after injection.
Data exclusions	N/A
Reproducibility	Unless otherwise indicated three replicates were generated to obtain results with sufficient accuracy for the study.
Randomization	N/A
Blinding	N/A

Did the study involve field work? ☐ Yes ☒ No

Reporting for specific materials, systems and methods

We require information from authors about some types of materials, experimental systems and methods used in many studies. Here, indicate whether each material, system or method listed is relevant to your study. If you are not sure if a list item applies to your research, read the appropriate section before selecting a response.

Materials & experimental systems

n/a	Involved in the study
☒	☐ Antibodies
☒	☐ Eukaryotic cell lines
☒	☐ Palaeontology
☒	☐ Animals and other organisms
☒	☐ Human research participants
☒	☐ Clinical data

Methods

n/a	Involved in the study
☒	☐ ChIP-seq
☒	☐ Flow cytometry
☒	☐ MRI-based neuroimaging
